# Nivolumab Plus Ipilimumab vs Nivolumab Alone in Advanced Cancers Other Than Melanoma: A Meta-Analysis

**DOI:** 10.1001/jamaoncol.2023.3295

**Published:** 2023-08-31

**Authors:** Anthony V. Serritella, Niraj K. Shenoy

**Affiliations:** 1 Robert H. Lurie Comprehensive Cancer Center of Northwestern University, Chicago, Illinois; 2 Northwestern University Feinberg School of Medicine, Chicago, Illinois

## Abstract

**Importance:**

Although the combination of nivolumab plus ipilimumab has unquestionable benefit over nivolumab monotherapy in advanced melanoma, currently no summative analyses have compared the combination with nivolumab monotherapy for advanced cancers other than melanoma.

**Objective:**

To examine whether the addition of ipilimumab to standard-dose nivolumab safely improves clinical outcomes in patients with advanced cancers other than melanoma.

**Data Sources:**

Electronic databases (PubMed, EBSCO Information Services, Embase, and Cochrane Library) were systematically searched for studies of standard-dose nivolumab plus ipilimumab vs nivolumab alone in the treatment of advanced cancers other than melanoma published from database inception to October 31, 2022.

**Study Selection:**

Eight studies (total patients, 1727; nivolumab plus ipilimumab group, 854; nivolumab monotherapy group, 873) met the selection criteria. Patients had squamous cell lung cancer, non–small cell lung cancer with programmed death ligand 1 level of 1% or higher, small cell lung cancer, pleural mesothelioma, urothelial carcinoma, esophagogastric carcinoma, sarcoma, or glioblastoma multiforme.

**Data Extraction and Synthesis:**

For comparison of overall survival (OS) and progression-free survival (PFS) outcomes, estimation of log(hazard ratios [HRs]) and SEs was initially performed for OS and PFS of each included study based on summary statistics extracted from individual Kaplan-Meier curves. Inverse-variance weighting was then used to compute pooled HRs (95% CIs). For comparison of dichotomous data (treatment-related grade 3 to 4 adverse events and discontinuations), odds ratios (ORs) were used, and the Mantel-Haenszel method was used to estimate pooled ORs (95% CIs).

**Results:**

Treatment with nivolumab plus ipilimumab was not associated with improvement in OS over treatment with nivolumab alone (pooled HR, 0.95; 95% CI, 0.85-1.06; *P* = .36), with 4 of the 8 studies having numerically lower median OS with the combination. Nivolumab plus ipilimumab combination therapy was associated with marginal, but not clinically meaningful, improvement in PFS over nivolumab alone (pooled HR, 0.88; 95% CI, 0.79-0.98; *P* = .02). The combination was associated with substantially higher treatment-related grade 3 to 4 adverse events (pooled OR, 1.84; 95% CI, 1.47-2.31; *P* < .001) and treatment-related discontinuations (pooled OR, 1.96; 95% CI, 1.44-2.65; *P* < .001). This finding was recapitulated in meta-analyses of individual grade 3 to 4 adverse events of hepatotoxicity, gastrointestinal toxicity, pneumonitis, endocrine dysfunction, dermatitis, fatigue.

**Conclusions and Relevance:**

In this meta-analysis of 8 advanced cancers other than melanoma, the differences detected in OS and PFS between nivolumab plus ipilimumab and nivolumab were not clinically meaningful (even though statistical significance was detected in PFS). Treatment-related higher-grade toxicity and discontinuations were substantially higher with the combination therapy. The data indicate that investigations of anti–programmed death 1 (PD1) plus anti–cytotoxic T-lymphocyte–associated protein 4 (CTLA-4) therapies in any nonmelanoma advanced cancer should be conducted along with anti-PD1 monotherapy to ensure that the net effect of the addition of anti-CTLA-4 to anti-PD1 can be clearly established for that cancer and setting and that unnecessary CTLA-4 inhibition with related toxic effects (both clinical and financial) can be avoided.

## Key Points

**Question:**

Does combination therapy of standard-dose nivolumab and ipilimumab improve clinical outcomes and justify additional toxicity compared with nivolumab monotherapy in advanced cancers other than metastatic melanoma?

**Findings:**

In this meta-analysis of 8 clinical trials including 1727 patients, the addition of ipilimumab to standard-dose nivolumab was not associated with a clinically meaningful improvement in overall survival or progression-free survival over standard-dose nivolumab alone. The combination was associated with substantially higher treatment-related high-grade toxicities without commensurate clinical benefit.

**Meaning:**

Combination therapy with nivolumab and ipilimumab may be unnecessary in many patient populations, and nivolumab or other anti–programmed death 1–directed therapy alone may deliver equivalent clinical outcomes with lower toxicity (clinical and financial) in many advanced cancers other than melanoma.

## Teaser

This meta-analysis investigates the efficacy and safety of nivolumab plus ipilimumab vs nivolumab alone in the treatment of advanced cancers other than melanoma.

## Introduction

Although the combination of nivolumab and ipilimumab has clearly demonstrated improvement in progression-free survival (PFS) and overall survival (OS) over nivolumab alone in metastatic melanoma (particularly in programmed death ligand 1 [PD-L1]–negative and *BRAF* [OMIM 164757] mutation–positive melanoma),^1,2^ its comparative efficacy over nivolumab monotherapy in other advanced cancers has not been well established. Relatively few trials to date have directly compared nivolumab plus ipilimumab with nivolumab monotherapy, and currently no summative analyses, to our knowledge, have compared the combination with nivolumab monotherapy for advanced cancers other than melanoma. However, the combination is often assumed superior to nivolumab monotherapy across cancers, and multiple trials have investigated the combination against the standard of care in different cancers. We thus sought to perform a meta-analysis aimed at investigating the efficacy and safety of nivolumab plus ipilimumab vs nivolumab alone in advanced cancers other than melanoma. For this meta-analysis, we included studies in which ipilimumab was added to standard-dose nivolumab (3 mg/kg or 240 mg).

## Methods

This meta-analysis conformed to the Preferred Reporting Items for Systematic Reviews and Meta-analyses (PRISMA) reporting guidelines. PubMed, EBSCO Information Services, Embase, and Cochrane Library were systematically searched for studies of standard-dose nivolumab plus ipilimumab vs nivolumab alone for the treatment of advanced cancers other than melanoma published from database inception to October 31, 2022. Full details of the search strategy, eligibility and inclusion criteria, outcome measures, and data extraction are described in eAppendix 1 in Supplement 1. The search process is outlined in the PRISMA schema (eFigure 1 in Supplement 1) and described in eAppendix 1 Supplement 1.

For comparison of time-to-event OS and PFS outcomes, estimation of log(hazard ratios [HRs]) and SEs was initially performed for OS and PFS of each included study based on summary statistics extracted from individual Kaplan-Meier (K-M) curves (incorporating number at risk, estimated number of events, and number censored for each specified interval, ensuring that the K-M curves generated from these numbers matched the published K-M curves), as described by Tierney et al.^3^ Detailed calculations for each study have been provided (eAppendix 3 in Supplement 1). Inverse-variance weighting was then used to compute pooled HRs (95% CIs) using the estimated individual log(HR) and SE values.

For comparison of dichotomous data, odds ratios (ORs) were used; all results were reported with 95% CIs. The Mantel-Haenszel method was used to estimate pooled ORs (95% CIs). The *I*^2^ test was used to assess impact of study heterogeneity (see eAppendix 1 in Supplement 1 for further details). RevMan, version 5.4 (Cochrane Collaboration) was used for the meta-analyses.

## Results

### Search Results and Study Characteristics

Eight studies (total patients, 1727; nivolumab plus ipilimumab group, 854; nivolumab monotherapy group, 873) met the selection criteria. Patients had squamous cell lung cancer,^4^ non–small cell lung cancer with programmed death ligand 1 level of 1% or higher,^5^ small cell lung cancer,^6^ pleural mesothelioma,^7^ urothelial carcinoma,^8^ esophagogastric carcinoma,^9^ sarcoma,^10^ or glioblastoma multiforme (see eAppendix 2 in Supplement 1 for further details).^11^ A summary of the included trials and their characteristics is given in the Table.

**Table.** Characteristics of Trials Included in the Meta-Analysis

Source	Phase	Randomized	Open label	Rating	Disease	Total No. of patients (I/C)^a^	Intervention^b^	Control	Median age, y	
									I	C
Gettinger et al,^4^ 2021	3	Yes	Yes	1	Metastatic squamous cell lung cancer	252 (125/127)	Nivolumab, 3 mg/kg every 2 wk, plus ipilimumab, 1 mg/kg every 6 wk until progression	Nivolumab, 3 mg/kg every 2 wk	67.5	68.1
Hellmann et al,^5^ 2019	3	Yes	Yes	1	Metastatic or recurrent NSCLC with PD-L1 ≥ 1%	792 (396/ 396)	Nivolumab, 3 mg/kg every 2 wk, plus ipilimumab, 1 mg/kg every 6 wk until progression	Nivolumab, 240 mg every 2 wk	64	64
Scherpereel et al,^7^ 2019	2	Yes	Yes	2	Relapsed pleural mesothelioma	125 (62/63)	Nivolumab, 3 mg/kg every 2 wk, plus ipilimumab, 1 mg/kg every 6 wk until progression	Nivolumab, 3 mg/kg every 2 wk	71.2	72.3
Sharma et al,^8^ 2019	1/2	No	Yes	2	Metastatic urothelial carcinoma	182 (104/78)	Nivolumab, 3 mg/kg every 3 wk, plus ipilimumab, 1 mg/kg every 3 wk, for 4 doses followed by nivolumab, 3 mg/kg for every 2-week maintenance	Nivolumab, 3 mg/kg every 2 wk	63	65.5
Janjigian et al,^9^ 2018	1/2	No	Yes	2	Metastatic esophagogastric carcinoma	111 (52/59)	Nivolumab, 3 mg/kg every 3 wk, plus ipilimumab, 1 mg/kg every 3 wk, for 4 doses followed by nivolumab, 3 mg/kg for every 2-week maintenance	Nivolumab, 3 mg/kg every 2 wk	58	60
D’Angelo et al,^10^ 2018	2	Yes	Yes	2	Advanced sarcoma	83 (41/42)	Nivolumab, 3 mg/kg every 3 wk, plus ipilimumab, 1 mg/kg every 3 wk, for 4 doses followed by nivolumab, 3 mg/kg for every 2-week maintenance	Nivolumab, 3 mg/kg every 2 wk	57	56
Omuro, et al,^11^ 2018	1	No	Yes	2	Glioblastoma multiforme	30 (20/10)	Nivolumab, 3 mg/kg every 3 wk, plus ipilimumab, 1 mg/kg every 3 wk, for 4 doses followed by nivolumab, 3 mg/kg for every 2-week maintenance	Nivolumab, 3 mg/kg every 2 wk	60	58.5
Antonia, et al,^6^ 2016	1/2	No	Yes	2	Recurrent small cell lung cancer	152 (54/98)	Nivolumab, 3 mg/kg every 2 wk, plus ipilimumab, 1 mg/kg for every 3 wk, for 4 doses followed by nivolumab, 3 mg/kg for every 2-week maintenance	Nivolumab, 3 mg/kg every 2 wk	61	63

Abbreviations: I, intervention; C, control; NSCLC, non–small cell lung cancer; PD-L1 programmed death ligand 1.

^a^ Total patients includes patients in the ipilimumab and standard-dose nivolumab and standard-dose nivolumab arms and excludes other patients and arms.

^b^ Trials may have included other dosing arms but were excluded from this meta-analysis.

### Efficacy

**Figure 1.**
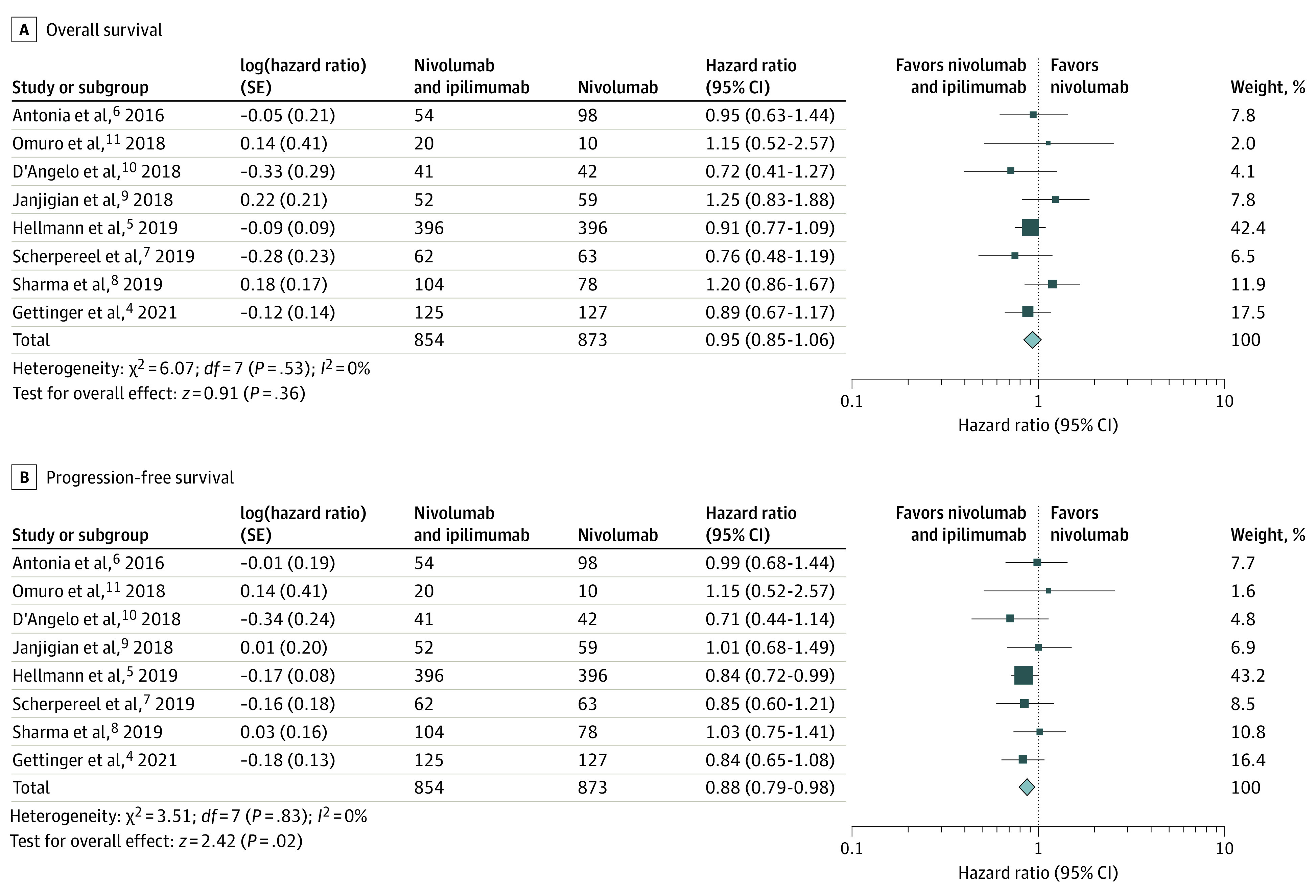
Meta-Analysis of Overall Survival and Progression-Free Survival With Nivolumab and Ipilimumab vs Nivolumab Alone in Advanced Cancers Other Than Melanoma A, Forest plot and meta-analysis of overall survival with nivolumab and ipilimumab vs nivolumab alone in advanced cancers other than melanoma show no improvement in overall survival with the combination (pooled hazard ratio, 0.95; 95% CI, 0.85-1.06; *P* = .36; *I*^2^ = 0%). B, Forest plot and meta-analysis of progression-free survival with nivolumab and ipilimumab vs nivolumab alone in advanced cancers other than melanoma show marginal, but not clinically meaningful, improvement in progression-free survival with the combination (pooled hazard ratio, 0.88; 95% CI, 0.79-0.98; *P* = .02; *I*^2^ = 0%).

The efficacy of nivolumab and ipilimumab vs nivolumab was evaluated by focusing on OS and PFS, the most clinically relevant metrics. Treatment with nivolumab and ipilimumab was not associated with an improvement in OS (pooled HR, 0.95; 95% CI, 0.85-1.06; *P* = .36; *I*^2^ = 0%) (Figure 1A). Of note, 4 of the studies^4,8,9,11^ had numerically lower median OS with the combination.

Nivolumab and ipilimumab combination therapy was associated with a marginal, but not clinically meaningful, improvement in PFS over nivolumab alone (pooled HR, 0.88; 95% CI, 0.79-0.98; *P* = .02; *I*^2^ = 0%) (Figure 1B). Of note, only 1 study^5^ had a statistically significant PFS benefit (HR, 0.84; 95% CI, 0.72-0.99), which was by far the largest study, with 792 patients, accounting for more than 40% of the weight in the meta-analysis. (A PFS HR of 0.88 [95% CI, 0.79-0.98] and an OS HR of 0.95 [95% CI, 0.85-1.06] falls well below what the American Society of Clinical Oncology considers a clinically meaningful incremental improvement for oncologic trial interventions, which is an OS HR of 0.8 or lower.^12^)

A sensitivity analysis was performed to determine the extent to which Hellmann et al^5^ influenced the OS and PFS meta-analysis (eFigure 2 in Supplement 1). The pooled HR without the study by Hellmann et al^5^ was 0.97 (95% CI, 0.84-1.13) for OS and 0.91 (95% CI, 0.79-1.04) for PFS (n = 936), revealing that without that study, the small PFS advantage dissipated across the other 936 patients in 7 studies.^4,6,7,8,9,10,11^ The sensitivity analysis did not affect study heterogeneity (*I*^2^ = 0%).

### Safety

**Figure 2.**
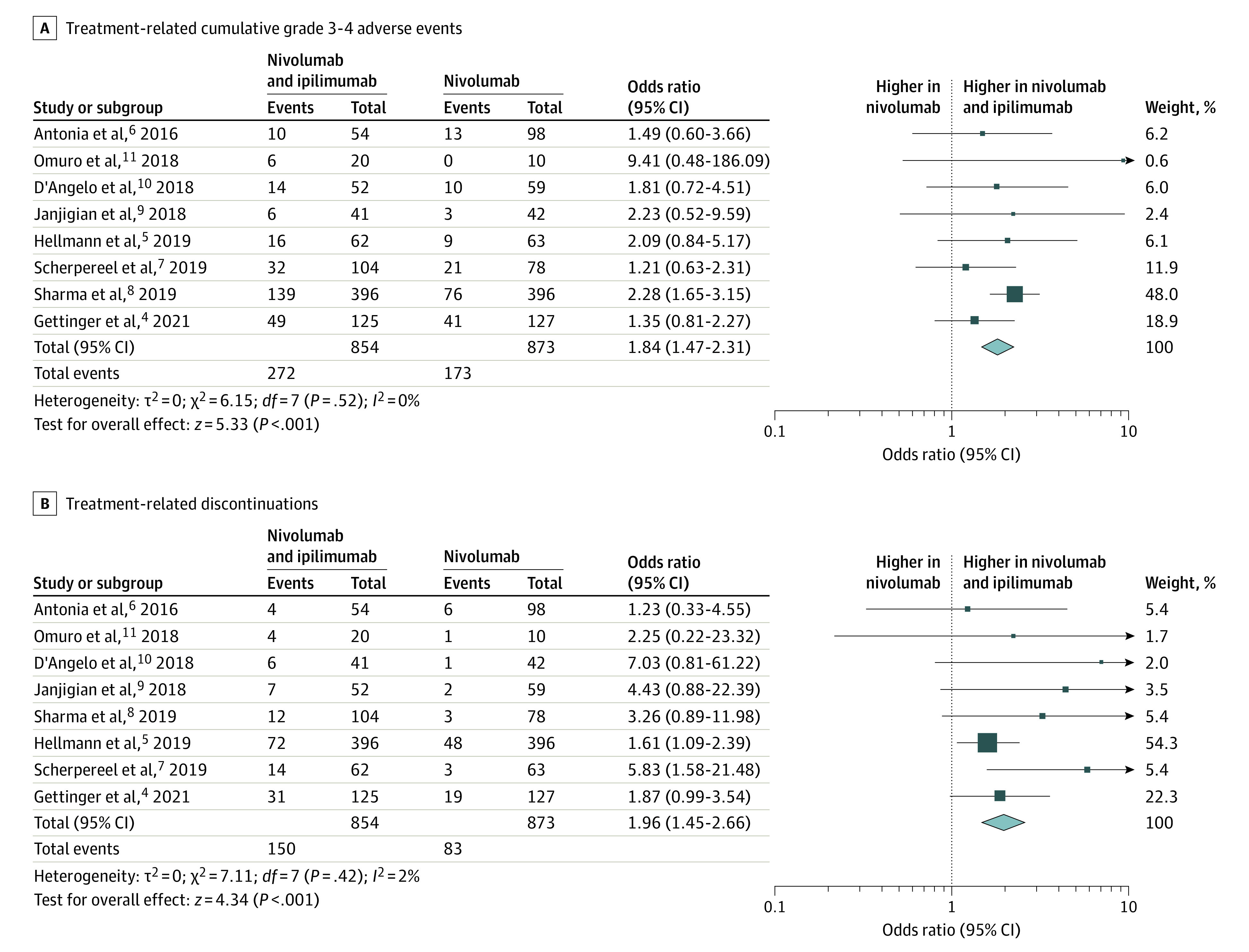
Meta-Analysis of Treatment-Related High-Grade Adverse Events and Discontinuations With Nivolumab and Ipilimumab vs Nivolumab Alone A, Forest plot and meta-analysis of cumulative grade 3 to 4 adverse events with nivolumab and ipilimumab vs nivolumab alone show substantial increase in severe adverse events with the combination (pooled odds ratio, 1.84; 95% CI, 1.47-2.31; *P* < .001; *I*^2^ = 0%). B, Forest plot and meta-analysis of treatment-related discontinuation with nivolumab and ipilimumab vs nivolumab alone show nearly 2 times greater odds of treatment-related discontinuation with the combination (pooled odds ratio, 1.96; 95% CI, 1.45-2.66; *P* < .001; *I*^2^ = 2%).

The pooled OR of grade 3 to 4 adverse events (AEs) for nivolumab and ipilimumab vs nivolumab and ipilimumab alone was 1.84 (95% CI, 1.47-2.31; *P* < .001; *I*^2^ = 0%) (Figure 2A). Across the 8 studies,^4,5,6,7,8,9,10,11^ there were 272 grade 3 to 4 AEs in 854 patients who received nivolumab and ipilimumab (approximately 0.47 odds) and 173 grade 3 to 4 AEs in 873 patients who received nivolumab alone (approximately 0.25 odds). Similarly, treatment-related discontinuations (Figure 2B) with nivolumab and ipilimumab vs nivolumab alone had a pooled OR of 1.96 (95% CI, 1.45-2.66; *P* < .001; *I*^2^ = 2%). Across all studies, 150 of 854 patients who received nivolumab and ipilimumab experienced treatment-related discontinuation (approximately 0.21 odds) compared with 83 of 873 patients who received nivolumab alone (approximately 0.11 odds).

The following grade 3 to 4 AEs occurred in the 854 patients in the combination treatment group: hepatotoxicity (n = 83 [9.7%]), gastrointestinal toxicity (n = 34 [4.0%]) pneumonitis (n = 31 [3.6%]), endocrine dysfunction (n = 29 [3.4%]), dermatitis (n = 30 [3.2%]), and fatigue (n = 30 [3.2%]). These grade 3 to 4 AEs were substantially lower in the 873 patients in the nivolumab group (hepatotoxicity [n = 26 (3.0%)], fatigue [n = 15 (1.7%)], pneumonitis [n = 13 (1.5%)], gastrointestinal toxicity [n = 9 (1%)], dermatitis [n = 8 (0.9%)], and endocrine dysfunction [n = 2 (0.2%)]). The pooled ORs for nivolumab and ipilimumab vs nivolumab for the individual grade 3 to 4 AEs were as follows: hepatotoxicity, 2.94 (95% CI, 1.67-5.15; *P* < .001); gastrointestinal toxicity, 3.28 (95% CI, 1.65-6.49; *P* < .001); pneumonitis, 2.37 (95% CI, 1.24-4.54; *P* = .009); endocrine dysfunction, 7.95 (95% CI, 2.57-24.65; *P* < .001); fatigue, 1.91 (95% CI, 1.03-3.52; *P* = .04); and dermatitis, 2.52 (95% CI, 0.68-9.36; *P* = .17) (eFigures 3-8 in Supplement 1).

## Discussion

In this meta-analysis of a wide spectrum of advanced cancers other than melanoma (small cell lung cancer, non–small cell lung cancer, squamous cell lung cancer, mesothelioma, urothelial carcinoma, esophagogastric carcinoma, sarcoma, and glioblastoma multiforme), the addition of ipilimumab to standard-dose nivolumab was not associated with a clinically meaningful improvement in OS or PFS over nivolumab monotherapy. Furthermore, the combination was associated with substantially higher treatment-related high-grade AEs and discontinuations.

Although 2 prior meta-analyses^13,14^ claimed that the combination of nivolumab and ipilimumab may be more effective than nivolumab alone for advanced cancers, these studies were heavily influenced by trials of advanced melanoma, accounting for more than 60% weight and driving up the pooled efficacy of nivolumab and ipilimumab compared with nivolumab. In addition, the meta-analyses^13,14^ reported a standard mean difference for survival outcomes, which is a much inferior statistical method for survival meta-analysis. Finally, additional trials investigating nivolumab and ipilimumab and nivolumab alone have since been reported, including 2 large randomized clinical trials in non–small cell lung cancer,^4,5^ included in our meta-analysis.

It is possible that certain immunogenic nonmelanoma cancers (such as renal cell carcinoma and triple-negative breast cancer) or specific subsets of populations (such as sarcomatoid renal cell carcinoma or squamous cell lung cancer with high tumor mutational burden and low PD-L1) may have survival benefit with the combination over single-agent nivolumab, although this remains to be determined. In advanced Merkel cell carcinoma, a rare immunogenic cutaneous cancer, nivolumab and ipilimumab combination therapy has demonstrated substantial benefit (objective response rate, 31%) even after prior anti-PD1/anti–PD-L1 exposure and a 100% objective response rate in the immune checkpoint inhibitor–naive setting, establishing the superiority of the combination in treating this cancer.^15^ Future randomized investigations in immunogenic cancers and biomarker-driven studies may identify combination-responsive cancers and subsets.

Our data indicate that investigations of anti-PD1 plus anti–cytotoxic T-lymphocyte–associated protein 4 (CTLA-4) therapies in any nonmelanoma advanced cancer should be conducted along with anti-PD1 monotherapy to ensure that the net effect of the addition of anti-CTLA-4 to anti-PD1 can be clearly established for that cancer and setting and that unnecessary CTLA-4 inhibition with related toxic effects (clinical and financial) can be avoided. Furthermore, in cancers in which nivolumab and ipilimumab combination therapy has been approved without comparison with nivolumab, noninferiority trials should be considered.

### Limitations

This study has some limitations. First, this meta-analysis includes several different tumor types, and there may be heterogeneity in the benefit of nivolumab and ipilimumab with relation to each tumor type. However, none of the trials individually had an OS benefit (with 4 of 8 having numerically lower median OS), and only 1 trial^5^ demonstrated a marginal PFS benefit for nivolumab and ipilimumab over nivolumab alone. Second, we did not include studies with a dosing regimen of 1 mg/kg of nivolumab and 3 mg/kg of ipilimumab to avoid further heterogeneity, and outcomes could possibly be different with this dosing regimen in certain disease contexts (as demonstrated in urothelial and esophagogastric carcinoma), although this regimen is known to be even more toxic than the more common regimen of 3 mg/kg of nivolumab and 1 mg/kg of ipilimumab, with substantially higher grade 3 to 4 event rate. Third, 1 study^5^ accounted for more than 40% of the analyses. However, sensitivity analysis without that study did not affect heterogeneity (*I*^2^ = 0) and revealed no benefit in OS or PFS for nivolumab and ipilimumab vs nivolumab across the other 936 patients in 7 studies.^4,6,7,8,9,10,11^

## Conclusion

In this meta-analysis of 8 clinical trials^4,5,6,7,8,9,10,11^ of 1727 patients with advanced cancers other than melanoma, the addition of ipilimumab to standard-dose nivolumab was not associated with a clinically meaningful improvement in OS or PFS over standard-dose nivolumab alone. The combination was associated with substantially higher treatment-related high-grade AEs and discontinuations. These findings suggest that combination therapy with nivolumab and ipilimumab may be unnecessary in many patient populations and that nivolumab alone may deliver equivalent clinical outcomes with lower toxicity (clinical and financial) in many advanced cancers other than melanoma.

## Notes

Supplement 1. **eAppendix 1.** Supplemental Methods **eAppendix 2.** Supplemental Results **eAppendix 3.** PFS and OS HR Calculation Analysis of Individual Studies **eFigure 1.** PRISMA Schema **eFigure 2.** OS and PFS Meta-analysis Without Hellman et al 2019 (Sensitivity Analysis) **eFigure 3.** Grade 3 to 4 Hepatotoxicity **eFigure 4.** Grade 3 to 4 GI Toxicity **eFigure 5.** Grade 3 to 4 Pneumonitis **eFigure 6.** Grade 3 to 4 Endocrine Dysfunction **eFigure 7.** Grade 3 to 4 Fatigue **eFigure 8.** Grade 3 to 4 Dermatitis

Supplement 2.
Data Sharing Statement
