# Supplementary material for: Nivolumab Plus Ipilimumab vs Nivolumab Alone in Advanced Cancers Other Than Melanoma: A Meta-Analysis
Source: JAMA Oncol. 2023 Aug 31;9(10):1441–6. doi: 10.1001/jamaoncol.2023.3295 (PMC10472261; doi:10.1001/jamaoncol.2023.3295)
Supplement: Supplement 1. — eAppendix 1. Supplemental Methods eAppendix 2. Supplemental Results eAppendix 3. PFS and OS HR Calculation Analysis of Individual Studies eFigure 1. PRISMA Schema eFigure 2. OS and PFS Meta-analysis Without Hellman et al 2019 (Sensitivity Analysis) eFigure 3. Grade 3 to 4 Hepatotoxicity eFigure 4. Grade 3 to 4 GI Toxicity eFigure 5. Grade 3 to 4 Pneumonitis eFigure 6. Grade 3 to 4 Endocrine Dysfunction eFigure 7. Grade 3 to 4 Fatigue eFigure 8. Grade 3 to 4 Dermatitis [file jamaoncol-e233295-s001.pdf]

Supplementary Online Content

Serritella AV, Shenoy NK. Nivolumab plus ipilimumab vs nivolumab alone in advanced cancers other than melanoma: a meta-analysis. *JAMA Oncol.* Published online August 31, 2023.
doi:10.1001/jamaoncol.2023.3295

eAppendix 1. Supplemental Methods

eAppendix 2. Supplemental Results

eAppendix 3. PFS and OS HR Calculation Analysis of Individual Studies

eFigure 1. PRISMA Schema

eFigure 2. OS and PFS Meta-analysis Without Hellman et al 2019 (Sensitivity Analysis)

eFigure 3. Grade 3 to 4 Hepatotoxicity

eFigure 4. Grade 3 to 4 GI Toxicity

eFigure 5. Grade 3 to 4 Pneumonitis

eFigure 6. Grade 3 to 4 Endocrine Dysfunction

eFigure 7. Grade 3 to 4 Fatigue

eFigure 8. Grade 3 to 4 Dermatitis

This supplementary material has been provided by the authors to give readers additional information about their work.

© 2023 Serritella AV et al. *JAMA Oncology*.

eAppendix 1. Supplemental Methods

Search Strategy

We searched PubMed, EBSCO Information Services, Embase, and The Cochrane Library databases for relevant English-language papers and abstracts that had been published by October 31, 2022. The following search terms were used: "nivolumab plus ipilimumab versus nivolumab." We also performed a manual search to find applicable studies in the references and related citations.

Eligibility and Inclusion Criteria

We included studies that fulfilled the following criteria: (a) **population**: patients with metastatic/advanced solid malignancies, excluding metastatic/advanced melanoma; (b) **intervention**: the following dose regimens ("nivo3 ipi1") were allowed: nivo 3 mg/kg every 2 weeks plus ipi 1mg/kg every 6 weeks until progression; nivo 3 mg/kg every 3 weeks plus ipi 1 mg/kg every 3 weeks for four doses followed by nivo 3 mg/kg every 2 weeks maintenance (c) **control**: the following doses were allowed: nivo monotherapy at a dose of 3 mg/kg every 2 weeks; flat dosing of nivo 240 mg every 2 weeks; (d) **prospective studies**- phase I, I/II, II or III clinical trials.

Studies of advanced/ metastatic melanoma were excluded. We also excluded studies or arms within studies that had combination dosing of nivo+ipi at doses other than as defined as nivo 3 ipi 1 as above. For example, excluded doses include nivo 1 mg/kg every 2 weeks plus ipi at a dose of 3 mg/kg every 6 weeks. Nivo1 ipi3 is a dosing regimen that is known to be even more toxic than nivo3 ipi1 and is approved in only one malignancy outside of melanoma thus far (hepatocellular carcinoma), so was excluded from the analysis for homogeneity.

Outcome Measures

The primary outcome measures were overall survival (OS), progression free survival (PFS), grade 3 or 4 adverse events (AEs), and treatment related discontinuations. Detailed information about grade 3-4 adverse events were also extracted and compared including hepatotoxicity, gastrointestinal toxicity, pneumonitis, endocrine dysfunction, dermatitis, and fatigue.

Data Extraction and Synthesis

Two reviewers independently screened the titles and abstracts of the retrieved citations. Discrepancies were resolved by discussions. A standardized extraction form was prepared using Microsoft Excel. The extracted data included first author, year of publication, study design, patient population, trial phase, study title, treatments, patients in each treatment arm, total patients in the study, median patient age by treatment arm, eastern cooperative oncology group (ECOG) performance status of patients by arm, median OS, PFS with 95% CI, treatment related discontinuations and detailed information on grade 3-4 adverse events. Summary statistics of Kaplan-Meier curves (OS and PFS) were extracted separately, also using Microsoft Excel.

© 2023 Serritella AV et al. *JAMA Oncology*.

‘difference in restricted mean survival time’ (dRMST)- based meta-analysis; the robustness of log-rank even with small departures from proportional hazards; the included studies not having major departures; the high ‘event rate’ in each included study given the patient population of advanced malignancies, put together, led to the determination that estimation of HR (and ln(HR), SE) for each individual study with K-M summary statistics followed by computing the pooled HR with inverse variance weighting (using the RevMan software) would be the more robust method overall for this meta-analysis. Extreme care was taken in the estimation of number of events and number censored for each specified time interval (along with the published number at risk), ensuring that the K-M curves generated from these numbers matched the published K-M curves, as explained in Tierney et al. Trials, 2007 (ref 4). The snips of calculation spreadsheets for both OS and PFS HR and the K-M curves generated have been provided for each included study (as a separate supplement file), for transparency and to enable comparison to the published K-M curves.

The estimation of pooled OR for dichotomous data (treatment-related high-grade adverse events and treatment-related discontinuations) using the Mantel-Haenszel method was also performed using the RevMan software.

Assessment of heterogeneity:

The I-square (I2) test was used to assess impact of study heterogeneity. None of the analyses had an $I^2 > 50\%$ (i.e., no severe heterogeneity) and the main outcome measures (OS, PFS, grade 3-4 AE, treatment-related discontinuations) had an $I^2 \leq 2\%$; therefore, the fixed effect model was chosen for all analyses.

© 2023 Serritella AV et al. *JAMA Oncology*.

eAppendix 2. Supplemental Results

Search Results and Study Characteristics

The initial search identified 168 publications. After exclusion of duplicates and unrelated publications, 57 publications remained. Of these, 36 were discarded after reading the titles and abstracts. After assessing the full text, 13 reports were further excluded and 8 studies were included for data analysis. (Process outlined in the PRISMA schema in **Figure S1)**. While 3 of the studies included treatment arms dosing both nivo 3 ipi 1 as well as nivo 1 ipi 3 (Sharma, 2019; Janjigian, 2018; Antonia, 2016; Omuro, 2017), we only included data from the nivo 3 ipi 1 arms as described above. The control group of nivo monotherapy was either 3 mg/kg or 240 mg every 2 weeks.

© 2023 Serritella AV et al. *JAMA Oncology*.

eAppendix 3. PFS and OS HR Calculation Analysis of Individual Studies

Hellmann et al 2019_ PFS

Key

- A: Checks
- B: Time interval
- C: Start of t
- D: Survival prob at t_{i-1} (%)
- E: Number alive at t_{i-1}
- F: Effective number at risk during t_{i-1},t_i
- G: Effective number events during t_{i-1},t_i
- H: Effective number censored during t_{i-1},t_i
- I: Survival prob at t_{i-1} (%)
- J: Number alive at t_{i-1}
- K: Effective number at risk during t_{i-1},t_i
- L: Effective number events during t_{i-1},t_i
- M: Effective number censored during t_{i-1},t_i
- N: Proportion of events in research arm
- O: Difference in events
- P: Variance of the log hazard ratio for t_k
- Q: Log hazard ratio for t_k
- R: Reciprocal of the variance of the log hazard ratio for t_k

HR	0.84
ln(HR)	-0.17
se(ln(HR))	0.08

A Checks	B $t_{i,i-1}$	C t_{i-1}	D $s_{2,i}$	E $n_{2,i}$	F $\bar{n}_{2,i}$	G $\bar{d}_{2,i}$	H $\bar{c}_{2,i}$	I $s_{1,i}$	J $n_{1,i}$	K $\bar{n}_{1,i}$	L $\bar{d}_{1,i}$	M $\bar{c}_{1,i}$	N $\bar{e}_{2,k}$	O $\bar{d}_{2,k}-\bar{e}_{2,k}$	P v_k	Q $ln(HR)_k$	R $1/v_k$
			Research(2): Nivo+Ipi					Control(1): Nivo					Outcomes				
	Total					294.0	102.0				314.0	82.0		-25.7	150.9		
	0 to 3	0	100	396	382.5	148.0	27.0	100	396	381.5	168.0	29.0	158.2	-10.2	79.01	-0.13	0.01
	3 to 6	3	61	221	217.5	56.0	7.0	56	199	194.5	54.0	9.0	58.1	-2.1	27.42	-0.08	0.04
	6 to 9	6	46	158	157.0	26.0	2.0	40	136	135.0	30.0	2.0	30.1	-4.1	13.91	-0.29	0.07
	9 to 12	9	38	130	127.5	17.0	5.0	31	104	103.5	18.0	1.0	19.3	-2.3	8.66	-0.27	0.12
	12 to 15	12	33	108	106.0	13.0	4.0	26	85	83.0	13.0	4.0	14.6	-1.6	6.42	-0.25	0.16
	15 to 18	15	29	91	90.5	7.0	1.0	22	68	67.5	11.0	1.0	10.3	-3.3	4.42	-0.75	0.23
	18 to 21	18	27	83	83.0	10.0	0.0	18	56	56.0	9.0	0.0	11.3	-1.3	4.56	-0.30	0.22
	21 to 24	21	23	73	71.5	5.0	3.0	15	47	46.0	3.0	2.0	4.8	0.1	1.90	0.07	0.53
	24 to 27	24	22	65	64.5	2.0	1.0	14	42	40.5	2.0	3.0	2.4	-0.5	0.94	-0.51	1.07
	27 to 30	27	21	62	57.0	5.0	10.0	14	37	33.0	5.0	8.0	6.3	-1.3	2.31	-0.58	0.43
	30 to 33	30	19	47	40.5	3.0	13.0	12	24	19.5	0.0	9.0	2.0	1.0	0.65	1.48	1.54
	33 to 36	33	18	31	20.0	2.0	22.0	12	15	9.5	1.0	11.0	2.0	0.0	0.65	-0.04	1.54
	36 to 39	36	16	7	3.5	0.0	7.0	10	3	1.5	0.0	3.0	0.0	0.0	0.00	na	na
	39 onwards	39	16	0.0	na	na	na	10	0.0	na	na	na	na	na	na	na	na
					na	na	na			na	na	na	na	na	na	na	na
					na	na	na			na	na	na	na	na	na	na	na
					na	na	na			na	na	na	na	na	na	na	na
					na	na	na			na	na	na	na	na	na	na	na
					na	na	na			na	na	na	na	na	na	na	na
					na	na	na			na	na	na	na	na	na	na	na
					na	na	na			na	na	na	na	na	na	na	na
					na	na	na			na	na	na	na	na	na	na	na
					na	na	na			na	na	na	na	na	na	na	na
					na	na	na			na	na	na	na	na	na	na	na
					na	na	na			na	na	na	na	na	na	na	na

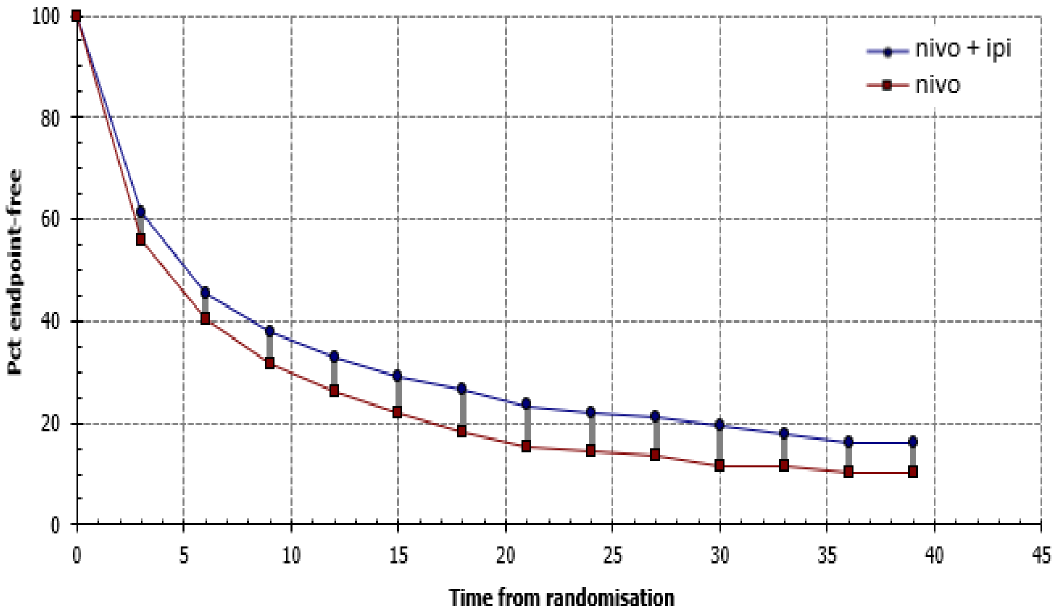

© 2023 Serritella AV et al. *JAMA Oncology*.

Hellmann et al 2019_ OS

Key

A: Checks
B: Time interval
C: Start of t

D: Survival prob at t_{i-1} (%)
E: Number alive at t_{i-1}
F: Effective number at risk during t_{i-1},t_i
G: Effective number events during t_{i-1},t_i
H: Effective number censored during t_{i-1},t_i

I: Survival prob at t_{i-1} (%)
J: Number alive at t_{i-1}
K: Effective number at risk during t_{i-1},t_i
L: Effective number events during t_{i-1},t_i
M: Effective number censored during t_{i-1},t_i

N: Proportion of events in research arm
O: Difference in events
P: Variance of the log hazard ratio for t_k
Q: Log hazard ratio for t_k
R: Reciprocal of the variance of the log hazard ratio for t_k

**HR**	**0.91**
**ln(HR)**	-0.10
**se(ln(HR))**	0.09

			Research(2): Nivo+Ipi					Control(1): Nivo					Outcomes				
	Total					258.0	138.0				274.0	122.0		-13.1	132.9		
A Checks	B $t_{i,i-1}$	C t_{i-1}	D $s_{2,i}$	E $n_{2,i}$	F $\bar{n}_{2,i}$	G $\bar{d}_{2,i}$	H $\bar{c}_{2,i}$	I $s_{1,i}$	J $n_{1,i}$	K $\bar{n}_{1,i}$	L $\bar{d}_{1,i}$	M $\bar{c}_{1,i}$	N $\bar{e}_{2,k}$	O $\bar{o}_{2,k}-\bar{e}_{2,k}$	P v_k	Q ln(HR)$_k$	R 1/v_k
	0 to 3	0	100	396	395.5	54.0	1.0	100	396	394.5	63.0	3.0	58.6	-4.6	29.25	-0.16	0.03
	3 to 6	3	86	341	340.0	44.0	2.0	84	330	329.5	30.0	1.0	37.6	6.4	18.50	0.35	0.05
	6 to 9	6	75	295	294.5	30.0	1.0	76	299	298.0	32.0	2.0	30.8	-0.8	15.49	-0.05	0.06
	9 to 12	9	68	264	263.0	18.0	2.0	68	265	265.0	45.0	0.0	31.4	-13.4	15.75	-0.85	0.06
	12 to 15	12	63	244	243.0	30.0	2.0	57	220	220.0	19.0	0.0	25.7	4.3	12.22	0.35	0.08
	15 to 18	15	55	212	211.5	21.0	1.0	52	201	201.0	25.0	0.0	23.6	-2.6	11.51	-0.22	0.09
	18 to 21	18	50	190	189.5	24.0	1.0	45	176	176.0	23.0	0.0	24.4	-0.4	11.74	-0.03	0.09
	21 to 24	21	43	165	165.0	12.0	0.0	39	153	152.5	13.0	1.0	13.0	-1.0	6.25	-0.16	0.16
	24 to 27	24	40	153	152.0	6.0	2.0	36	139	138.5	9.0	1.0	7.9	-1.9	3.75	-0.50	0.27
	27 to 30	27	39	145	142.0	10.0	6.0	34	129	125.0	6.0	8.0	8.5	1.5	3.99	0.37	0.25
	30 to 33	30	36	129	111.5	3.0	35.0	32	115	94.0	3.0	42.0	3.3	-0.2	1.51	-0.16	0.66
	33 to 36	33	35	91	68.0	4.0	46.0	31	70	55.0	4.0	30.0	4.4	-0.4	1.96	-0.22	0.51
	36 to 39	36	33	41	26.0	2.0	30.0	29	36	23.9	1.9	24.1	2.0	0.0	0.96	-0.03	1.04
	39 to 42	39	30	9.0	5.0	0.0	8.0	27	10.0	6.0	0.0	8.0	0.0	0.0	0.00	na	na
	42 to 45	42	30	1.0	0.5	0.0	1.0	27	2.0	1.0	0.0	2.0	0.0	0.0	0.00	na	na
	45 onwards	45	30	0.0	na	na	na	27	0.0	na	na	na	na	na	na	na	na
					na	na	na			na	na	na	na	na	na	na	na
					na	na	na			na	na	na	na	na	na	na	na
					na	na	na			na	na	na	na	na	na	na	na
					na	na	na			na	na	na	na	na	na	na	na
					na	na	na			na	na	na	na	na	na	na	na
					na	na	na			na	na	na	na	na	na	na	na
					na	na	na			na	na	na	na	na	na	na	na
					na	na	na			na	na	na	na	na	na	na	na
					na	na	na			na	na	na	na	na	na	na	na

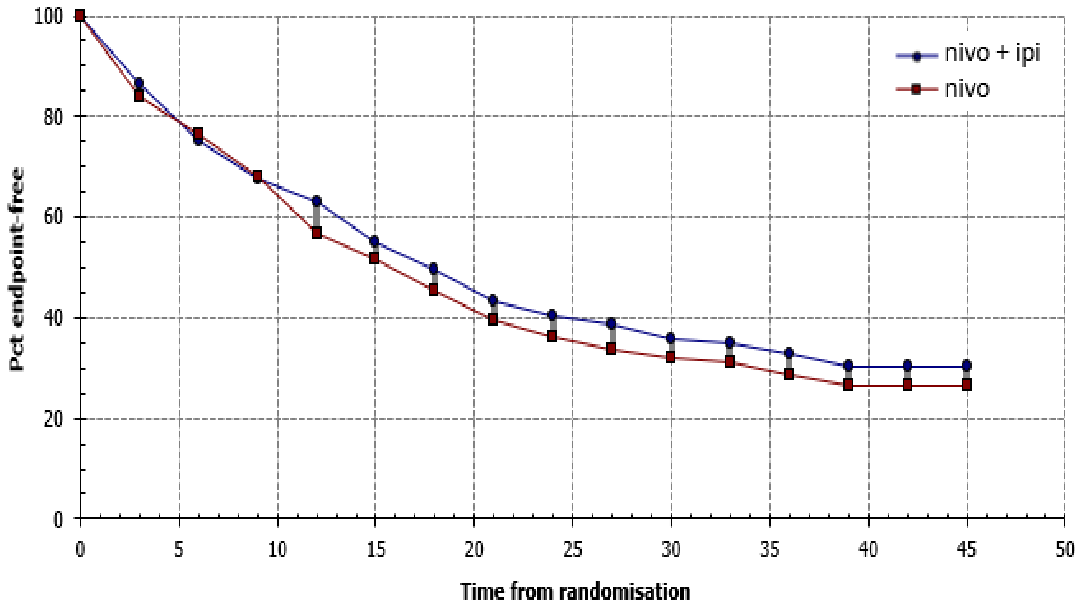

© 2023 Serritella AV et al. *JAMA Oncology*.

Gettinger et al 2021_ PFS

Key

A: Checks
B: Time interval
C: Start of t

D: Survival prob at t_{i-1} (%)
E: Number alive at t_{i-1}
F: Effective number at risk during t_{i-1}, t_i
G: Effective number events during t_{i-1}, t_i
H: Effective number censored during t_{i-1}, t_i

I: Survival prob at t_{i-1} (%)
J: Number alive at t_{i-1}
K: Effective number at risk during t_{i-1}, t_i
L: Effective number events during t_{i-1}, t_i
M: Effective number censored during t_{i-1}, t_i

N: Proportion of events in research arm
O: Difference in events
P: Variance of the log hazard ratio for t_k
Q: Log hazard ratio for t_k
R: Reciprocal of the variance of the log hazard ratio for t_k

HR	0.84
ln(HR)	-0.18
se(ln(HR))	0.13

			Research(2): Nivo+Ipi					Control(1): Nivo					Outcomes				
	Total					110.0	15.0				122.0	5.0		-10.1	57.4		
A Checks	B $t_{i,i-1}$	C t_{i-1}	D $s_{2,i}$	E $n_{2,i}$	F $n'_{2,i}$	G $d'_{2,i}$	H $c'_{2,i}$	I $s_{1,i}$	J $n_{1,i}$	K $n'_{1,i}$	L $d'_{1,i}$	M $c'_{1,i}$	N $e'_{2,k}$	O $e'_{2,k}-e'_{2}$	P v_k	Q $ln(HR)_k$	R $1/v_k$
	0 to 6	0	100	125	124.5	85.0	1.0	100	127	126.0	95.0	2.0	89.5	-4.5	45.01	-0.10	0.02
	6 to 12	6	32	39	38.0	17.0	2.0	25	30	30.0	18.0	0.0	19.6	-2.5	8.63	-0.29	0.12
	12 to 18	12	18	20	20.0	4.0	0.0	10	12	12.0	3.0	0.0	4.3	-0.3	1.63	-0.21	0.61
	18 to 24	18	14	16	14.0	2.0	4.0	7	9	8.0	3.0	2.0	3.2	-1.2	1.17	-1.03	0.86
	24 to 30	24	12	10	9.5	0.0	1.0	5	4	4.0	1.0	0.0	0.7	-0.7	0.21	-3.38	4.80
	30 to 36	30	12	9	6.5	2.0	5.0	3	3	2.5	2.0	1.0	2.9	-0.9	0.80	-1.11	1.26
	36 to 42	36	8	2	1.0	0.0	2.0	1	0	0.0	0.0	0.0	0.0	0.0	0.00	na	na
Problem--->	42 onwards	42	8	0	na	na	na		0	na	na	na	na	na	na	na	na
					na	na	na			na	na	na	na	na	na	na	na
					na	na	na			na	na	na	na	na	na	na	na
					na	na	na			na	na	na	na	na	na	na	na
					na	na	na			na	na	na	na	na	na	na	na
					na	na	na			na	na	na	na	na	na	na	na
					na	na	na			na	na	na	na	na	na	na	na
					na	na	na			na	na	na	na	na	na	na	na
					na	na	na			na	na	na	na	na	na	na	na
					na	na	na			na	na	na	na	na	na	na	na
					na	na	na			na	na	na	na	na	na	na	na
					na	na	na			na	na	na	na	na	na	na	na
					na	na	na			na	na	na	na	na	na	na	na
					na	na	na			na	na	na	na	na	na	na	na
					na	na	na			na	na	na	na	na	na	na	na
					na	na	na			na	na	na	na	na	na	na	na
					na	na	na			na	na	na	na	na	na	na	na
					na	na	na			na	na	na	na	na	na	na	na

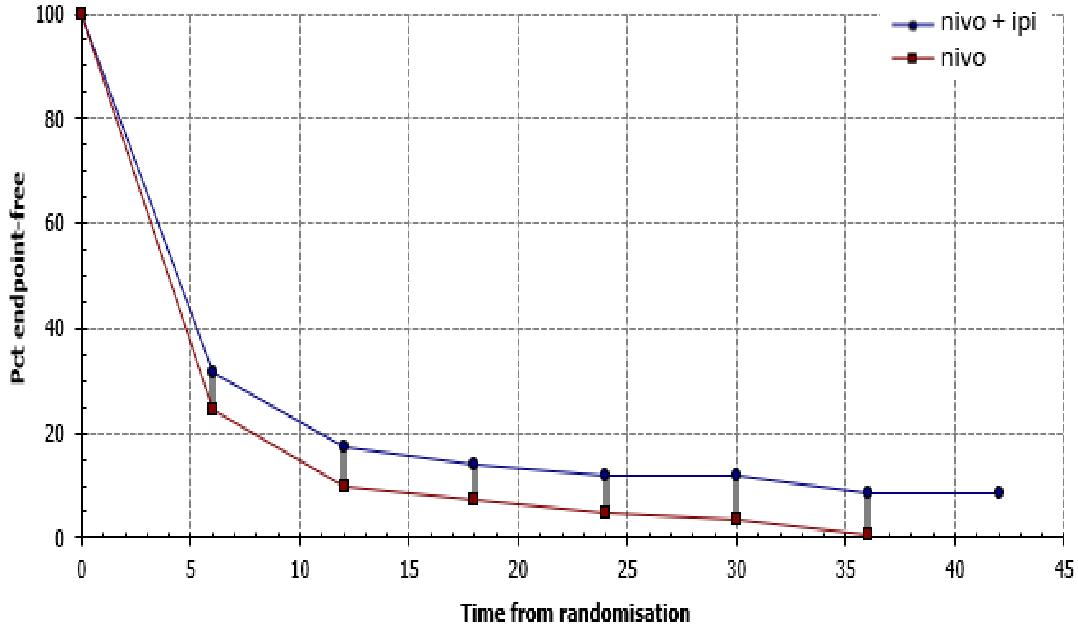

© 2023 Serritella AV et al. *JAMA Oncology*.

Gettinger et al 2021_ OS

Key

A: Checks
B: Time interval
C: Start of t
D: Survival prob at t_{i-1} (%)
E: Number alive at t_{i-1}
F: Effective number at risk during t_{i-1}, t_i
G: Effective number events during t_{i-1}, t_i
H: Effective number censored during t_{i-1}, t_i
I: Survival prob at t_{i-1} (%)
J: Number alive at t_{i-1}
K: Effective number at risk during t_{i-1}, t_i
L: Effective number events during t_{i-1}, t_i
M: Effective number censored during t_{i-1}, t_i
N: Proportion of events in research arm
O: Difference in events
P: Variance of the log hazard ratio for t_k
Q: Log hazard ratio for t_k
R: Reciprocal of the variance of the log hazard ratio for t_k

HR	0.89
ln(HR)	-0.12
se(ln(HR))	0.14

			Research(2): Nivo+Ipi					Control(1): Nivo					Outcomes				
	Total					92.0	33.0				105.0	22.0		-5.6	49.1		
A	B	C	D	E	F	G	H	I	J	K	L	M	N	O	P	Q	R
Checks	$t_{i,i-1}$	t_{i-1}	$s_{2,i}$	$n_{2,i}$	$\bar{n}_{2,i}$	$\bar{d}_{2,i}$	$\bar{c}_{2,i}$	$s_{1,i}$	$n_{1,i}$	$\bar{n}_{1,i}$	$\bar{d}_{1,i}$	$\bar{c}_{1,i}$	$\bar{e}_{2,k}$	$\bar{o}_{2,k}-\bar{e}_{2,k}$	v_k	$\ln(HR)_k$	$1/v_k$
	0 to 6	0	100	125	124.5	43.0	1.0	100	127	126.0	37.0	2.0	39.8	3.3	20.00	0.16	0.05
	6 to 12	6	65	81	80.5	25.0	1.0	71	88	87.5	33.0	1.0	27.8	-2.8	14.47	-0.19	0.07
	12 to 18	12	45	55	54.0	13.0	2.0	44	54	53.5	13.0	1.0	13.1	0.0	6.51	-0.01	0.15
	18 to 24	18	34	40	36.0	7.0	8.0	33	40	39.0	14.0	2.0	10.1	-3.1	5.24	-0.59	0.19
	24 to 30	24	28	25	23.5	2.0	3.0	21	24	19.5	6.0	9.0	4.4	-2.3	1.99	-1.18	0.50
	30 to 36	30	25	20	14.5	2.0	11.0	15	9	7.0	1.0	4.0	2.0	0.0	0.64	-0.03	1.56
	36 to 42	36	22	7	3.5	0.0	7.0	13	4	2.5	1.0	3.0	0.6	-0.6	0.25	-2.39	3.96
	42 onwards	42	22	0	na	na	na	8	0	na	na	na	na	na	na	na	na
					na	na	na			na	na	na	na	na	na	na	na
					na	na	na			na	na	na	na	na	na	na	na
					na	na	na			na	na	na	na	na	na	na	na
					na	na	na			na	na	na	na	na	na	na	na
					na	na	na			na	na	na	na	na	na	na	na
					na	na	na			na	na	na	na	na	na	na	na
					na	na	na			na	na	na	na	na	na	na	na
					na	na	na			na	na	na	na	na	na	na	na
					na	na	na			na	na	na	na	na	na	na	na
					na	na	na			na	na	na	na	na	na	na	na
					na	na	na			na	na	na	na	na	na	na	na
					na	na	na			na	na	na	na	na	na	na	na
					na	na	na			na	na	na	na	na	na	na	na
					na	na	na			na	na	na	na	na	na	na	na
					na	na	na			na	na	na	na	na	na	na	na
					na	na	na			na	na	na	na	na	na	na	na
					na	na	na			na	na	na	na	na	na	na	na

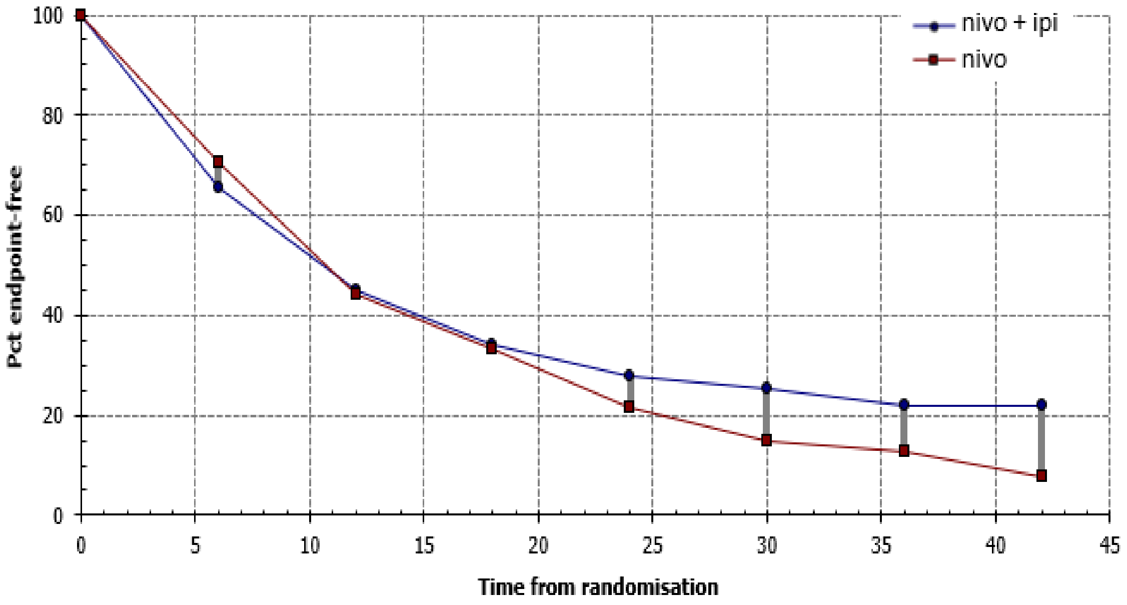

© 2023 Serritella AV et al. *JAMA Oncology*.

Sharma et al 2019_ PFS

Key

A: Checks
B: Time interval
C: Start of t

D: Survival prob at t_{i-1} (%)
E: Number alive at t_{i-1}
F: Effective number at risk during t_{i-1},t_i
G: Effective number events during t_{i-1},t_i
H: Effective number censored during t_{i-1},t_i

I: Survival prob at t_{i-1} (%)
J: Number alive at t_{i-1}
K: Effective number at risk during t_{i-1},t_i
L: Effective number events during t_{i-1},t_i
M: Effective number censored during t_{i-1},t_i

N: Proportion of events in research arm
O: Difference in events
P: Variance of the log hazard ratio for t_k
Q: Log hazard ratio for t_k
R: Reciprocal of the variance of the log hazard ratio for t_k

HR	1.03
ln(HR)	0.03
se(ln(HR))	0.16

			Research(2): Nivo+Ipi					Control(1): Nivo					Outcomes				
	Total					89.0	15.0				68.0	10.0		1.0	38.3		
A	B	C	D	E	F	G	H	I	J	K	L	M	N	O	P	Q	R
Checks	$t_{i,i-1}$	t_{i-1}	$s_{2,i}$	$n_{2,i}$	$\bar{n}_{2,i}$	$\bar{d}_{2,i}$	$\bar{c}_{2,i}$	$s_{1,i}$	$n_{1,i}$	$\bar{n}_{1,i}$	$\bar{d}_{1,i}$	$\bar{c}_{1,i}$	$\bar{e}_{2,k}$	$\bar{o}_{2,k}-\bar{e}_{2,k}$	v_k	$\ln(HR)_k$	$1/v_k$
	0 to 3	0	100	104	102.5	59.0	3.0	100	78	78.0	44.0	0.0	58.5	0.5	25.28	0.02	0.04
	3 to 6	3	42	42	41.5	11.0	1.0	44	34	33.5	6.0	1.0	9.4	1.6	4.19	0.39	0.24
	6 to 9	6	31	30	30.0	8.0	0.0	36	27	26.5	6.0	1.0	7.4	0.6	3.48	0.17	0.29
	9 to 12	9	23	22	22.0	1.0	0.0	28	20	19.5	7.0	1.0	4.2	-3.2	1.99	-1.63	0.50
	12 to 15	12	22	21	21.0	3.0	0.0	18	12	12.0	1.0	0.0	2.5	0.4	0.93	0.48	1.08
	15 to 18	15	19	18	18.0	0.0	0.0	16	11	11.0	0.0	0.0	0.0	0.0	0.00	na	na
	18 to 21	18	19	18	18.0	1.0	0.0	16	11	11.0	0.0	0.0	0.6	0.4	0.22	1.61	4.46
	21 to 24	21	18	17	17.0	3.0	0.0	16	11	10.5	0.0	1.0	1.8	1.1	0.70	1.62	1.43
	24 to 27	24	15	14	14.0	1.0	0.0	16	10	9.5	0.0	1.0	0.6	0.4	0.24	1.68	4.25
	27 to 30	27	14	13	13.0	0.0	0.0	16	9	9.0	1.0	0.0	0.6	-0.6	0.24	-2.44	4.16
	30 to 33	30	14	13	12.5	0.0	1.0	15	8	8.0	0.0	0.0	0.0	0.0	0.00	na	na
	33 to 36	33	14	12	11.5	2.0	1.0	15	8	8.0	1.0	0.0	1.8	0.2	0.73	0.34	1.37
	36 to 39	36	11	9	5.5	0.0	7.0	13	7	6.5	1.0	1.0	0.5	-0.5	0.26	-1.84	3.84
	39 to 42	39	11	2.0	1.5	0.0	1.0	11	5.0	4.0	0.0	2.0	0.0	0.0	0.00	na	na
	42 to 44	42	11	1.0	0.5	0.0	1.0	11	3.0	2.5	0.0	1.0	0.0	0.0	0.00	na	na
	44 to 46	44	11	0.0	0.0	0.0	0.0	11	2.0	1.5	1.0	1.0	0.0	0.0	0.00	na	na
Problem--->	46 onwards	46		0.0	na	na	na	4	0.0	na	na	na	na	na	na	na	na
					na	na	na			na	na	na	na	na	na	na	na
					na	na	na			na	na	na	na	na	na	na	na
					na	na	na			na	na	na	na	na	na	na	na
					na	na	na			na	na	na	na	na	na	na	na
					na	na	na			na	na	na	na	na	na	na	na
					na	na	na			na	na	na	na	na	na	na	na
					na	na	na			na	na	na	na	na	na	na	na
					na	na	na			na	na	na	na	na	na	na	na

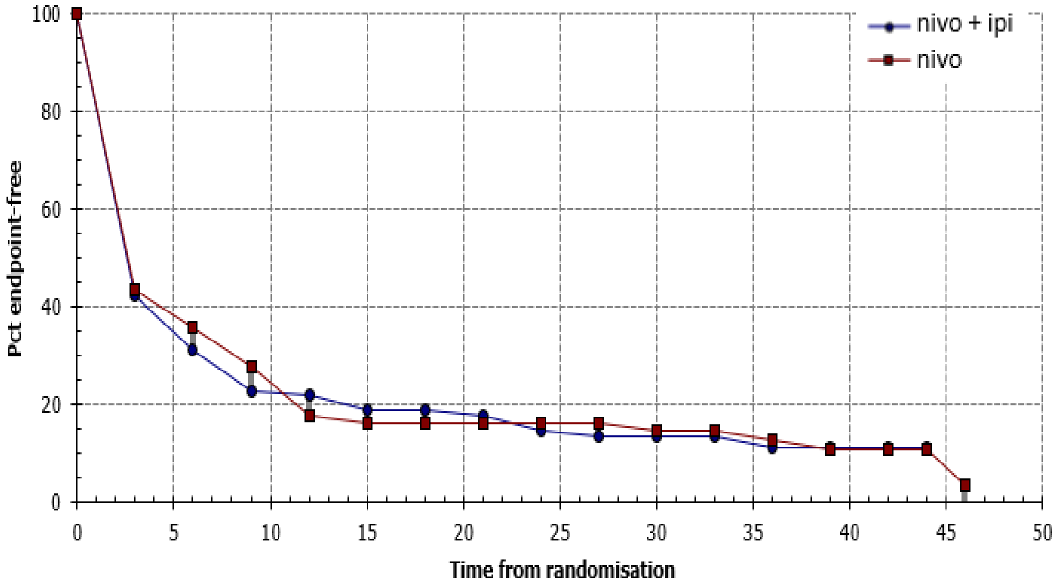

© 2023 Serritella AV et al. *JAMA Oncology*.

Sharma et al 2019_ OS

Key

- **A:** Checks
- **B:** Time interval
- **C:** Start of t
- **D:** Survival prob at t_{i-1} (%)
- **E:** Number alive at t_{i-1}
- **F:** Effective number at risk during t_{i-1},t_i
- **G:** Effective number events during t_{i-1},t_i
- **H:** Effective number censored during t_{i-1},t_i
- **I:** Survival prob at t_{i-1} (%)
- **J:** Number alive at t_{i-1}
- **K:** Effective number at risk during t_{i-1},t_i
- **L:** Effective number events during t_{i-1},t_i
- **M:** Effective number censored during t_{i-1},t_i
- **N:** Proportion of events in research arm
- **O:** Difference in events
- **P:** Variance of the log hazard ratio for t_k
- **Q:** Log hazard ratio for t_k
- **R:** Reciprocal of the variance of the log hazard ratio for t_k

**HR**	1.20
**ln(HR)**	0.18
**se(ln(HR))**	0.17

			Research(2): Nivo+Ipi					Control(1): Nivo					Outcomes				
	Total					81.0	23.0				56.0	22.0		6.1	33.7		
A	B	C	D	E	F	G	H	I	J	K	L	M	N	O	P	Q	R
Checks	$t_{i,i-1}$	t_{i-1}	$s_{2,i}$	$n_{2,i}$	$\bar{n}_{2,i}$	$\bar{d}_{2,i}$	$\bar{c}_{2,i}$	$s_{1,i}$	$n_{1,i}$	$\bar{n}_{1,i}$	$\bar{d}_{1,i}$	$\bar{c}_{1,i}$	$\bar{e}_{2,k}$	$\bar{o}_{2,k}-\bar{e}_{2,k}$	v_k	$\ln(HR)_k$	$1/v_k$
	0 to 3	0	100	104	104.0	18.0	0.0	100	78	78.0	17.0	0.0	20.0	-2.0	8.57	-0.23	0.12
	3 to 6	3	83	86	86.0	25.0	0.0	78	61	61.0	7.0	0.0	18.7	6.3	7.77	0.81	0.13
	6 to 9	6	59	61	61.0	15.0	0.0	69	54	54.0	13.0	0.0	14.9	0.1	6.97	0.02	0.14
	9 to 12	9	44	46	45.5	6.0	1.0	53	41	40.5	4.0	1.0	5.3	0.7	2.48	0.30	0.40
	12 to 15	12	38	39	39.0	7.0	0.0	47	36	36.0	4.0	0.0	5.7	1.3	2.74	0.47	0.37
	15 to 18	15	32	32	32.0	1.0	0.0	42	32	31.5	1.0	1.0	1.0	0.0	0.50	0.03	2.01
	18 to 21	18	31	31	31.0	1.0	0.0	41	30	30.0	1.0	0.0	1.0	0.0	0.50	-0.01	1.99
	21 to 24	21	30	30	30.0	2.0	0.0	40	29	29.0	1.0	0.0	1.5	0.5	0.76	0.66	1.32
	24 to 27	24	28	28	28.0	2.0	0.0	38	28	28.0	4.0	0.0	3.0	-1.0	1.50	-0.64	0.67
	27 to 30	27	26	26	26.0	0.0	0.0	33	24	23.5	1.0	1.0	0.5	-0.5	0.24	-2.11	4.15
	30 to 33	30	26	26	24.5	1.0	3.0	31	22	22.0	0.0	0.0	0.5	0.5	0.24	1.90	4.18
	33 to 36	33	25	22	22.0	3.0	0.0	31	22	21.5	2.0	1.0	2.5	0.4	1.24	0.36	0.80
	36 to 39	36	21	19	17.0	0.0	4.0	29	19	17.5	0.0	3.0	0.0	0.0	0.00	na	na
	39 to 42	39	21	15.0	10.0	0.0	10.0	29	16.0	12.5	0.0	7.0	0.0	0.0	0.00	na	na
	42 to 45	42	21	5.0	2.5	0.0	5.0	29	9.0	7.1	1.1	3.9	0.3	-0.3	0.22	-1.35	4.65
	45 to 48	45	21	0.0	0.0	0.0	0.0	24	4.0	2.0	0.0	4.0	0.0	0.0	0.00	na	na
Problem--->	48 onwards	48		0.0	na	na	na	24	0.0	na	na	na	na	na	na	na	na
					na	na	na			na	na	na	na	na	na	na	na
					na	na	na			na	na	na	na	na	na	na	na
					na	na	na			na	na	na	na	na	na	na	na
					na	na	na			na	na	na	na	na	na	na	na
					na	na	na			na	na	na	na	na	na	na	na
					na	na	na			na	na	na	na	na	na	na	na
					na	na	na			na	na	na	na	na	na	na	na
					na	na	na			na	na	na	na	na	na	na	na

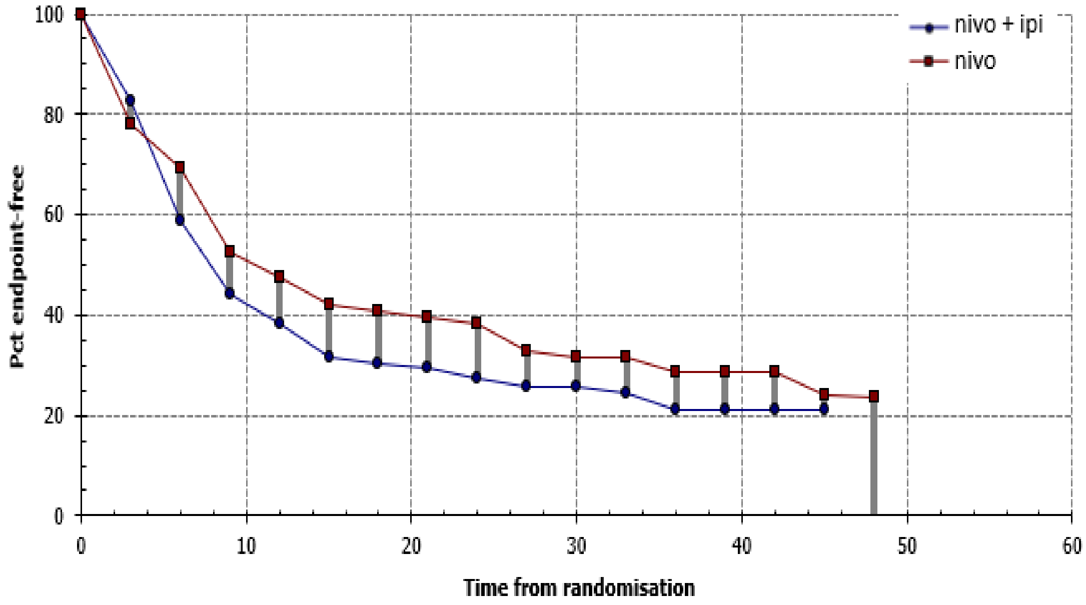

© 2023 Serritella AV et al. *JAMA Oncology*.

Janjigian et al 2018_ PFS

Key

A: Checks
B: Time interval
C: Start of t

D: Survival prob at t_{i-1} (%)
E: Number alive at t_{i-1}
F: Effective number at risk during t_{i-1},t_i
G: Effective number events during t_{i-1},t_i
H: Effective number censored during t_{i-1},t_i

I: Survival prob at t_{i-1} (%)
J: Number alive at t_{i-1}
K: Effective number at risk during t_{i-1},t_i
L: Effective number events during t_{i-1},t_i
M: Effective number censored during t_{i-1},t_i

N: Proportion of events in research arm
O: Difference in events
P: Variance of the log hazard ratio for t_k
Q: Log hazard ratio for t_k
R: Reciprocal of the variance of the log hazard ratio for t_k

**HR**	1.01
**ln(HR)**	0.01
**se(ln(HR))**	0.20

			Research(2): Nivo+Ipi					Control(1): Nivo					Outcomes				
	Total					48.0	4.0				51.0	8.0		0.2	24.5		
A	B	C	D	E	F	G	H	I	J	K	L	M	N	O	P	Q	R
Checks	$t_{i,i-1}$	t_{i-1}	$s_{2,i}$	$n_{2,i}$	$\bar{n}_{2,i}$	$\bar{d}_{2,i}$	$\bar{c}_{2,i}$	$s_{1,i}$	$n_{1,i}$	$\bar{n}_{1,i}$	$\bar{d}_{1,i}$	$\bar{c}_{1,i}$	$\bar{e}_{2,k}$	$\bar{O}_{2,k}-\bar{e}_{2}$	v_k	$\ln(HR)_k$	$1/v_k$
	0 to 3	0	100	52	51.0	36.0	2.0	100	59	57.0	40.0	4.0	35.9	0.1	18.95	0.01	0.05
	3 to 6	3	29	14	14.0	9.0	0.0	30	15	15.0	9.0	0.0	8.7	0.3	4.49	0.07	0.22
	6 to 9	6	11	5	5.0	1.0	0.0	12	6	5.0	1.0	2.0	1.0	0.0	0.50	0.04	1.99
	9 to 12	9	8	4	4.0	0.0	0.0	10	3	2.0	0.0	2.0	0.0	0.0	0.00	na	na
	12 to 15	12	8	4	4.0	2.0	0.0	10	1	1.0	0.0	0.0	1.6	0.4	0.32	1.25	3.15
	15 to 18	15	4	2	1.5	0.0	1.0	10	1	1.0	1.0	0.0	0.6	-0.6	0.24	-2.50	4.17
	18 to 21	18	4	1	0.5	0.0	1.0	0	0	0.0	0.0	0.0	0.0	0.0	0.00	na	na
	21 onwards	21	4	0	na	na	na	0	0	na	na	na	na	na	na	na	na
					na	na	na			na	na	na	na	na	na	na	na
					na	na	na			na	na	na	na	na	na	na	na
					na	na	na			na	na	na	na	na	na	na	na
					na	na	na			na	na	na	na	na	na	na	na
					na	na	na			na	na	na	na	na	na	na	na
					na	na	na			na	na	na	na	na	na	na	na
					na	na	na			na	na	na	na	na	na	na	na
					na	na	na			na	na	na	na	na	na	na	na
					na	na	na			na	na	na	na	na	na	na	na
					na	na	na			na	na	na	na	na	na	na	na
					na	na	na			na	na	na	na	na	na	na	na
					na	na	na			na	na	na	na	na	na	na	na
					na	na	na			na	na	na	na	na	na	na	na
					na	na	na			na	na	na	na	na	na	na	na
					na	na	na			na	na	na	na	na	na	na	na
					na	na	na			na	na	na	na	na	na	na	na
					na	na	na			na	na	na	na	na	na	na	na

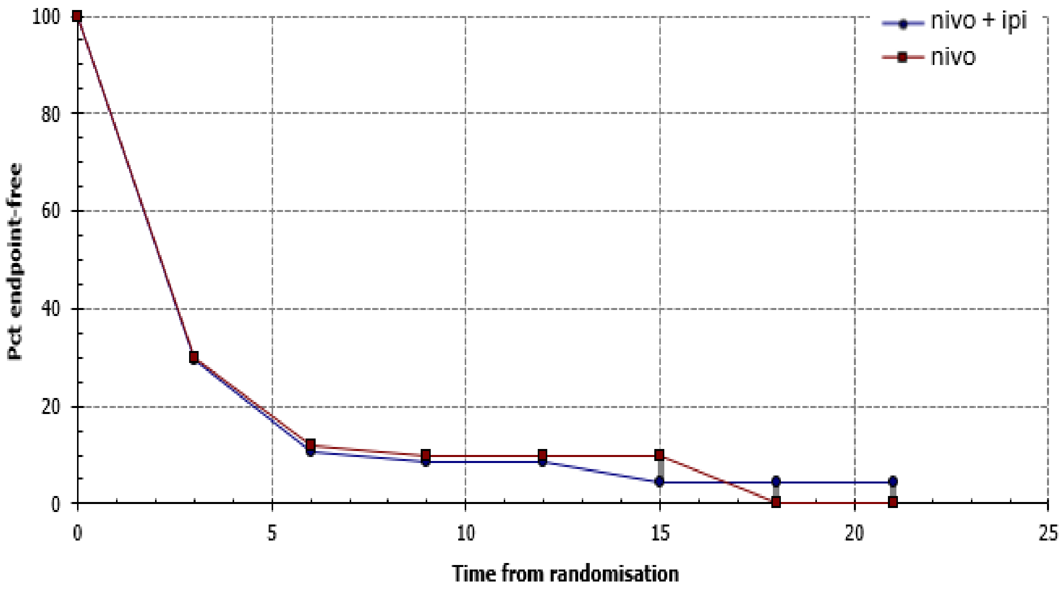

© 2023 Serritella AV et al. *JAMA Oncology*.

Janjigian et al. 2018_ OS

Key

A: Checks
B: Time interval
C: Start of t

D: Survival prob at t_{i-1} (%)
E: Number alive at t_{i-1}
F: Effective number at risk during t_{i-1},t_i
G: Effective number events during t_{i-1},t_i
H: Effective number censored during t_{i-1},t_i

I: Survival prob at t_{i-1} (%)
J: Number alive at t_{i-1}
K: Effective number at risk during t_{i-1},t_i
L: Effective number events during t_{i-1},t_i
M: Effective number censored during t_{i-1},t_i

N: Proportion of events in research arm
O: Difference in events
P: Variance of the log hazard ratio for t_k
Q: Log hazard ratio for t_k
R: Reciprocal of the variance of the log hazard ratio for t_k

HR	1.24
ln(HR)	0.22
se(ln(HR))	0.21

			Research(2): Nivo+Ipi					Control(1): Nivo					Outcomes				
	Total					46.0	6.0				49.0	10.0		4.9	22.8		
A Checks	B $t_{i,i-1}$	C t_{i-1}	D $s_{2,i}$	E $n_{2,i}$	F $\bar{n}_{2,i}$	G $\bar{d}_{2,i}$	H $\bar{c}_{2,i}$	I $s_{1,i}$	J $n_{1,i}$	K $\bar{n}_{1,i}$	L $\bar{d}_{1,i}$	M $\bar{c}_{1,i}$	N $\bar{e}_{2,k}$	O $\bar{o}_{2,k}-\bar{e}_{2,k}$	P v_k	Q $\ln(HR)_k$	R $1/v_k$
	0 to 3	0	100	52	52.0	19.0	0.0	100	59	59.0	19.0	0.0	17.8	1.2	9.46	0.13	0.11
	3 to 6	3	63	33	33.0	13.0	0.0	68	40	39.0	12.0	2.0	11.4	1.6	6.20	0.25	0.16
	6 to 9	6	38	20	20.0	2.0	0.0	47	26	26.0	5.0	0.0	3.0	-1.0	1.71	-0.60	0.58
	9 to 12	9	35	18	17.5	6.0	1.0	38	21	21.0	1.0	0.0	3.2	2.8	1.73	1.62	0.58
	12 to 15	12	23	11	11.0	3.0	0.0	36	20	20.0	5.0	0.0	2.8	0.2	1.84	0.11	0.54
	15 to 18	15	17	8	7.0	2.0	2.0	27	15	15.0	4.0	0.0	1.9	0.1	1.31	0.06	0.77
	18 to 21	18	12	4	3.5	0.0	1.0	20	11	9.0	2.0	4.0	0.6	-0.6	0.40	-1.39	2.49
	21 to 24	21	12	3	2.0	1.0	2.0	16	5	5.0	0.0	0.0	0.3	0.7	0.20	3.51	5.02
	24 to 27	24	6	0	na	na	na	16	5	5.0	1.0	0.0	na	na	na	na	na
Problem--->	27 to 30	27			na	na	na	13	4	2.6	0.1	2.9	na	na	na	na	na
Problem--->	30 to 33	30			na	na	na	12	1	0.5	0.0	1.0	na	na	na	na	na
Problem--->	33 onwards	33			na	na	na	12	0	na	na	na	na	na	na	na	na
					na	na	na			na	na	na	na	na	na	na	na
					na	na	na			na	na	na	na	na	na	na	na
					na	na	na			na	na	na	na	na	na	na	na
					na	na	na			na	na	na	na	na	na	na	na
					na	na	na			na	na	na	na	na	na	na	na
					na	na	na			na	na	na	na	na	na	na	na
					na	na	na			na	na	na	na	na	na	na	na
					na	na	na			na	na	na	na	na	na	na	na
					na	na	na			na	na	na	na	na	na	na	na
					na	na	na			na	na	na	na	na	na	na	na
					na	na	na			na	na	na	na	na	na	na	na
					na	na	na			na	na	na	na	na	na	na	na
					na	na	na			na	na	na	na	na	na	na	na

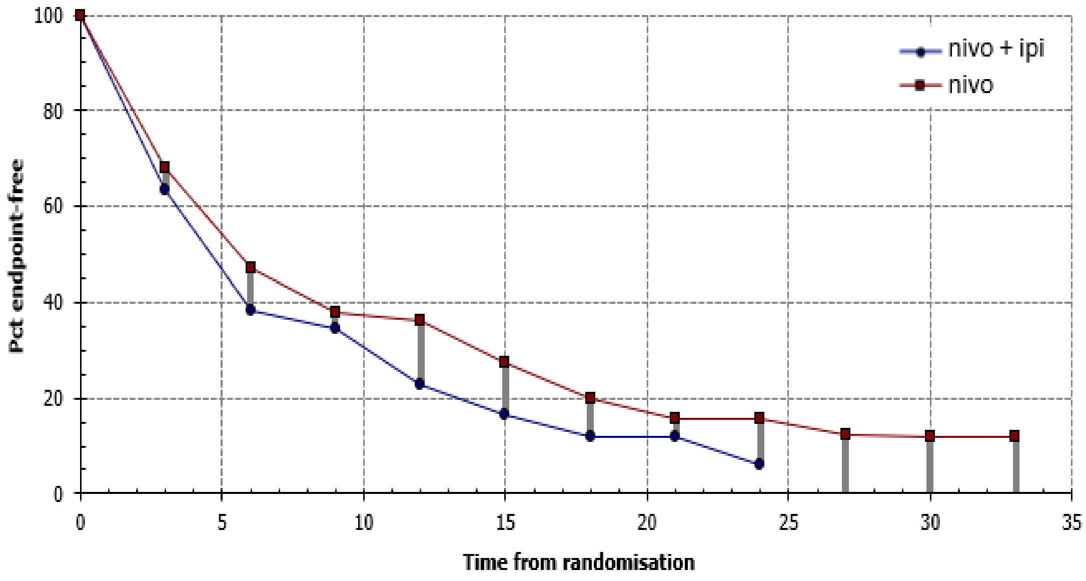

© 2023 Serritella AV et al. *JAMA Oncology*.

Scherpereel et al 2019_ PFS

Key

A: Checks
B: Time interval
C: Start of t

D: Survival prob at t_{i-1} (%)
E: Number alive at t_{i-1}
F: Effective number at risk during t_{i-1},t_i
G: Effective number events during t_{i-1},t_i
H: Effective number censored during t_{i-1},t_i

I: Survival prob at t_{i-1} (%)
J: Number alive at t_{i-1}
K: Effective number at risk during t_{i-1},t_i
L: Effective number events during t_{i-1},t_i
M: Effective number censored during t_{i-1},t_i

N: Proportion of events in research arm
O: Difference in events
P: Variance of the log hazard ratio for t_k
Q: Log hazard ratio for t_k
R: Reciprocal of the variance of the log hazard ratio for t_k

**HR**	**0.85**
**ln(HR)**	-0.16
**se(ln(HR))**	0.19

			Research(2): Nivo+Ipi					Control(1): Nivo					Outcomes				
	Total					53.0	9.0				57.0	6.0		-4.3	27.3		
A	B	C	D	E	F	G	H	I	J	K	L	M	N	O	P	Q	R
Checks	$t_{i,i-1}$	t_{i-1}	$s_{2,i}$	$n_{2,i}$	$\tilde{n}_{2,i}$	$\tilde{d}_{2,i}$	$\tilde{c}_{2,i}$	$s_{1,i}$	$n_{1,i}$	$\tilde{n}_{1,i}$	$\tilde{d}_{1,i}$	$\tilde{c}_{1,i}$	$\tilde{e}_{2,k}$	$\tilde{d}_{2,k}-\tilde{e}_{2,k}$	v_k	$ln(HR)_k$	$1/v_k$
	0 to 1	0	100	62	62.0	5.0	0.0	100	63	63.0	1.0	0.0	3.0	2.0	1.51	1.35	0.66
	1 to 2	1	92	57	57.0	3.0	0.0	98	62	62.0	7.0	0.0	4.8	-1.8	2.49	-0.72	0.40
	2 to 5	2	87	54	54.0	20.0	0.0	87	55	55.0	27.0	0.0	23.3	-3.3	11.75	-0.28	0.09
	5 to 10	5	55	34	34.0	15.0	0.0	44	28	28.0	15.0	0.0	16.5	-1.5	7.43	-0.20	0.13
	10 to 15	10	31	19	19.0	8.0	0.0	21	13	13.0	4.0	0.0	7.1	0.9	2.89	0.30	0.35
	15 to 20	15	18	11	7.5	2.0	7.0	14	9	6.5	3.0	5.0	2.7	-0.7	1.24	-0.54	0.81
	20 to 25	20	13	2	1.0	0.0	2.0	8	1	0.5	0.0	1.0	0.0	0.0	0.00	na	na
	25 onwards	25	13	0	na	na	na	8	0	na	na	na	na	na	na	na	na
					na	na	na			na	na	na	na	na	na	na	na
					na	na	na			na	na	na	na	na	na	na	na
					na	na	na			na	na	na	na	na	na	na	na
					na	na	na			na	na	na	na	na	na	na	na
					na	na	na			na	na	na	na	na	na	na	na
					na	na	na			na	na	na	na	na	na	na	na
					na	na	na			na	na	na	na	na	na	na	na
					na	na	na			na	na	na	na	na	na	na	na
					na	na	na			na	na	na	na	na	na	na	na
					na	na	na			na	na	na	na	na	na	na	na
					na	na	na			na	na	na	na	na	na	na	na
					na	na	na			na	na	na	na	na	na	na	na
					na	na	na			na	na	na	na	na	na	na	na
					na	na	na			na	na	na	na	na	na	na	na
					na	na	na			na	na	na	na	na	na	na	na
					na	na	na			na	na	na	na	na	na	na	na
					na	na	na			na	na	na	na	na	na	na	na

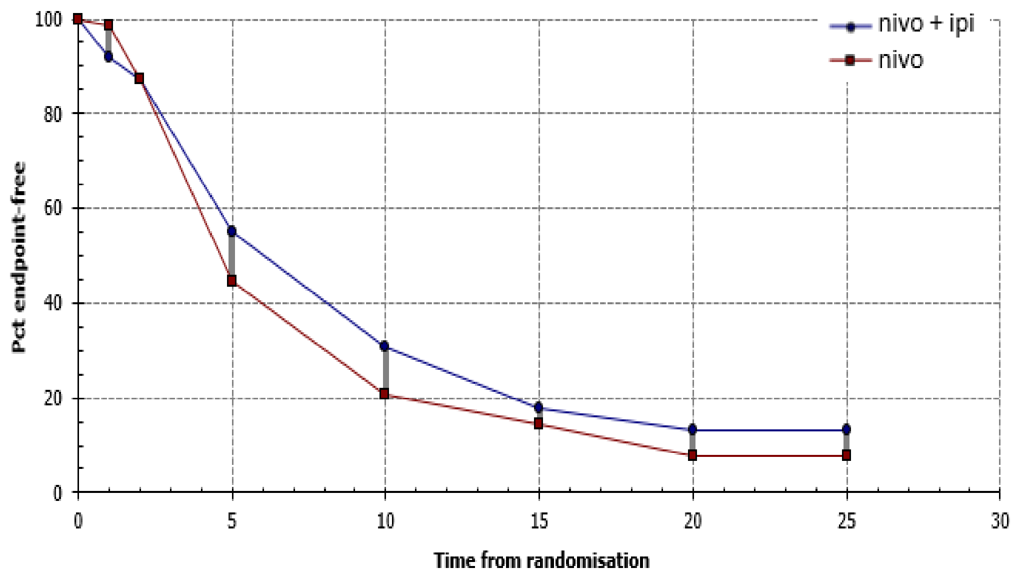

© 2023 Serritella AV et al. *JAMA Oncology*.

Scherpereel et al. 2019_ OS

Key

A: Checks
B: Time interval
C: Start of t
D: Survival prob at t_{i-1} (%)
E: Number alive at t_{i-1}
F: Effective number at risk during t_{i-1},t_i
G: Effective number events during t_{i-1},t_i
H: Effective number censored during t_{i-1},t_i
I: Survival prob at t_{i-1} (%)
J: Number alive at t_{i-1}
K: Effective number at risk during t_{i-1},t_i
L: Effective number events during t_{i-1},t_i
M: Effective number censored during t_{i-1},t_i
N: Proportion of events in research arm
O: Difference in events
P: Variance of the log hazard ratio for t_k
Q: Log hazard ratio for t_k
R: Reciprocal of the variance of the log hazard ratio for t_k

**HR**	0.76
**ln(HR)**	-0.28
**se(ln(HR))**	0.23

			Research(2): Nivo+Ipi						Control(1): Nivo						Outcomes				
	Total					32.0	30.0				41.0	22.0		-5.1	18.2		
A<br>Checks	B<br>$t_{i,i-1}$	C<br>t_{i-1}	D<br>$s_{2,i}$	E<br>$n_{2,i}$	F<br>$\bar{n}_{2,i}$	G<br>$\bar{d}_{2,i}$	H<br>$\bar{c}_{2,i}$	I<br>$s_{1,i}$	J<br>$n_{1,i}$	K<br>$\bar{n}_{1,i}$	L<br>$\bar{d}_{1,i}$	M<br>$\bar{c}_{1,i}$	N<br>$\bar{e}_{2,k}$	O<br>$\bar{e}_{2,k}-\bar{e}_2$	P<br>v_k	Q<br>$\ln(HR)_k$	R<br>$1/v_k$
	0 to 3	0	100	62	62.0	9.0	0.0	100	63	63.0	4.0	0.0	6.4	2.6	3.25	0.79	0.31
	3 to 5	3	85	53	53.0	4.0	0.0	94	59	59.0	15.0	0.0	9.0	-5.0	4.74	-1.05	0.21
	5 to 10	5	79	49	49.0	9.0	0.0	70	44	44.0	9.0	0.0	9.5	-0.5	4.49	-0.11	0.22
	10 to 13	10	65	40	40.0	7.0	0.0	56	35	35.0	4.0	0.0	5.9	1.1	2.74	0.41	0.37
	13 to 15	13	53	33	33.0	1.0	0.0	49	31	31.0	4.0	0.0	2.6	-1.6	1.25	-1.26	0.80
	15 to 20	15	52	32	21.0	2.0	22.0	43	27	18.5	5.0	17.0	3.7	-1.7	1.73	-1.00	0.58
	20 to 25	20	47	8	4.0	0.0	8.0	31	5	2.5	0.0	5.0	0.0	0.0	0.00	na	na
	25 onwards	25	47	0	na	na	na	31	0	na	na	na	na	na	na	na	na
					na	na	na			na	na	na	na	na	na	na	na
					na	na	na			na	na	na	na	na	na	na	na
					na	na	na			na	na	na	na	na	na	na	na
					na	na	na			na	na	na	na	na	na	na	na
					na	na	na			na	na	na	na	na	na	na	na
					na	na	na			na	na	na	na	na	na	na	na
					na	na	na			na	na	na	na	na	na	na	na
					na	na	na			na	na	na	na	na	na	na	na
					na	na	na			na	na	na	na	na	na	na	na
					na	na	na			na	na	na	na	na	na	na	na
					na	na	na			na	na	na	na	na	na	na	na
					na	na	na			na	na	na	na	na	na	na	na
					na	na	na			na	na	na	na	na	na	na	na
					na	na	na			na	na	na	na	na	na	na	na
					na	na	na			na	na	na	na	na	na	na	na
					na	na	na			na	na	na	na	na	na	na	na
					na	na	na			na	na	na	na	na	na	na	na

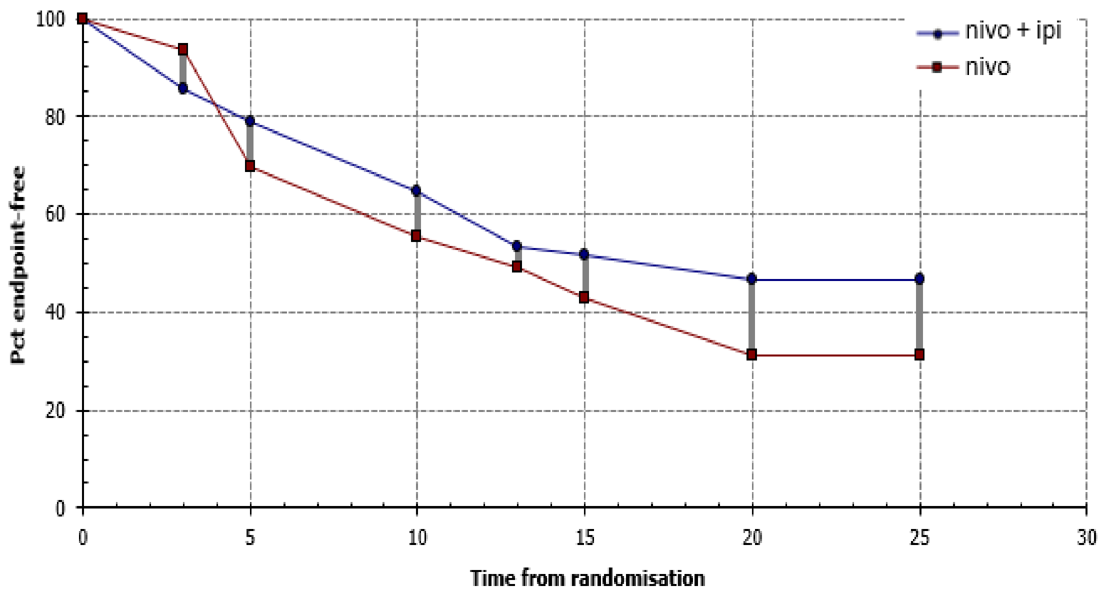

© 2023 Serritella AV et al. *JAMA Oncology*.

Antonia et al 2016_ PFS

Key

- **A:** Checks
- **B:** Time interval
- **C:** Start of t
- **D:** Survival prob at t_{i-1} (%)
- **E:** Number alive at t_{i-1}
- **F:** Effective number at risk during t_{i-1},t_i
- **G:** Effective number events during t_{i-1},t_i
- **H:** Effective number censored during t_{i-1},t_i
- **I:** Survival prob at t_{i-1} (%)
- **J:** Number alive at t_{i-1}
- **K:** Effective number at risk during t_{i-1},t_i
- **L:** Effective number events during t_{i-1},t_i
- **M:** Effective number censored during t_{i-1},t_i
- **N:** Proportion of events in research arm
- **O:** Difference in events
- **P:** Variance of the log hazard ratio for t_k
- **Q:** Log hazard ratio for t_k
- **R:** Reciprocal of the variance of the log hazard ratio for t_k

**HR**	**0.99**
**ln(HR)**	-0.01
**se(ln(HR))**	0.19

			Research(2): Nivo+Ipi					Control(1): Nivo					Outcomes				
	Total					42.0	12.0				76.0	22.0		-0.2	27.1		
A Checks	B $t_{i,i-1}$	C t_{i-1}	D $s_{2,i}$	E $n_{2,i}$	F $\bar{n}_{2,i}$	G $\bar{d}_{2,i}$	H $\bar{c}_{2,i}$	I $s_{1,i}$	J $n_{1,i}$	K $\bar{n}_{1,i}$	L $\bar{d}_{1,i}$	M $\bar{c}_{1,i}$	N $\bar{e}_{2,k}$	O $\bar{d}_{2,k}-\bar{e}_{2,k}$	P v_k	Q $ln(HR)_k$	R $1/v_k$
	0 to 1.5	0	100	54	53.0	33.0	2.0	100	98	95.0	53.0	6.0	30.8	2.2	19.78	0.11	0.05
	1.5 to 3	1.5	38	19	18.0	3.0	2.0	44	39	36.0	16.0	6.0	6.3	-3.3	4.22	-0.79	0.24
	3 to 6	3	31	14	10.5	4.0	7.0	25	17	14.5	6.0	5.0	4.2	-0.2	2.44	-0.07	0.41
	6 to 9	6	19	3	2.5	2.0	1.0	14	6	6.0	1.0	0.0	0.9	1.1	0.62	1.77	1.62
	9 to 12	9	4	0	na	na	na	12	5	4.5	0.0	1.0	na	na	na	na	na
Problem--->	12 to 15	12			na	na	na	12	4	3.5	0.0	1.0	na	na	na	na	na
Problem--->	15 to 18	15			na	na	na	12	3	3.0	0.0	0.0	na	na	na	na	na
Problem--->	18 to 21	18			na	na	na	12	3	1.5	0.0	3.0	na	na	na	na	na
Problem--->	21 onwards	21			na	na	na	12	0	na	na	na	na	na	na	na	na
					na	na	na			na	na	na	na	na	na	na	na
					na	na	na			na	na	na	na	na	na	na	na
					na	na	na			na	na	na	na	na	na	na	na
					na	na	na			na	na	na	na	na	na	na	na
					na	na	na			na	na	na	na	na	na	na	na
					na	na	na			na	na	na	na	na	na	na	na
					na	na	na			na	na	na	na	na	na	na	na
					na	na	na			na	na	na	na	na	na	na	na
					na	na	na			na	na	na	na	na	na	na	na
					na	na	na			na	na	na	na	na	na	na	na
					na	na	na			na	na	na	na	na	na	na	na
					na	na	na			na	na	na	na	na	na	na	na
					na	na	na			na	na	na	na	na	na	na	na
					na	na	na			na	na	na	na	na	na	na	na
					na	na	na			na	na	na	na	na	na	na	na
					na	na	na			na	na	na	na	na	na	na	na

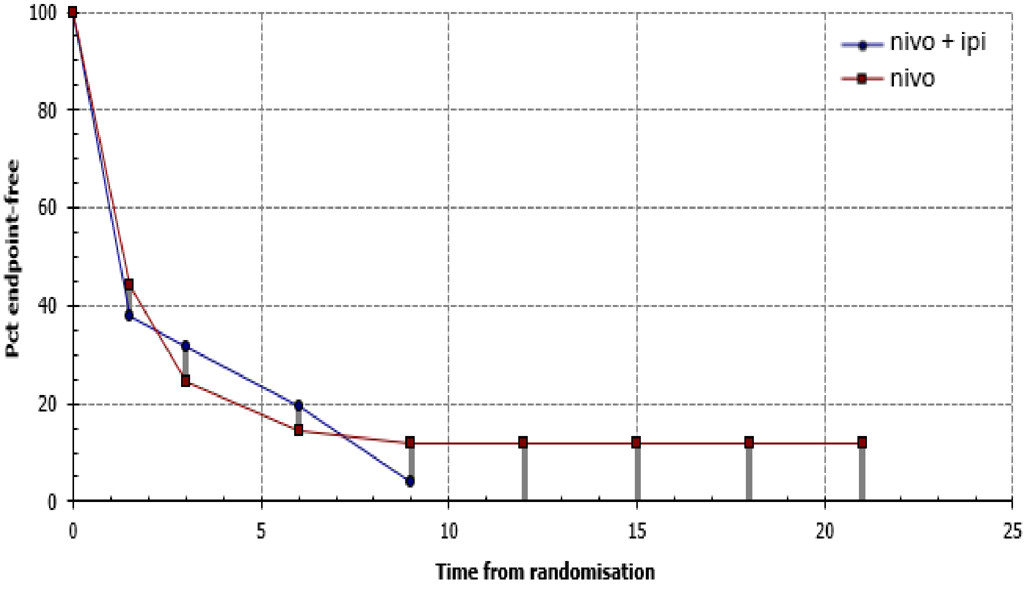

© 2023 Serritella AV et al. *JAMA Oncology*.

Antonia et al. 2016_ OS

Key

HR **0.95**
ln(HR) -0.05
se(ln(HR)) 0.21

A: Checks
B: Time interval
C: Start of t
D: Survival prob at t_{i-1} (%)
E: Number alive at t_{i-1}
F: Effective number at risk during t_{i-1}, t_i
G: Effective number events during t_{i-1}, t_i
H: Effective number censored during t_{i-1}, t_i
I: Survival prob at t_{i-1} (%)
J: Number alive at t_{i-1}
K: Effective number at risk during t_{i-1}, t_i
L: Effective number events during t_{i-1}, t_i
M: Effective number censored during t_{i-1}, t_i
N: Proportion of events in research arm
O: Difference in events
P: Variance of the log hazard ratio for t_k
Q: Log hazard ratio for t_k
R: Reciprocal of the variance of the log hazard ratio for t_k

			Research(2): Nivo+Ipi						Control(1): Nivo						Outcomes				
	Total					35.0	20.0				60.0	38.0		-1.2	22.0		
A Checks	B $t_{i,i-1}$	C t_{i-1}	D $s_{2,i}$	E $n_{2,i}$	F $\bar{n}_{2,i}$	G $\bar{d}_{2,i}$	H $\bar{c}_{2,i}$	I $s_{1,i}$	J $n_{1,i}$	K $\bar{n}_{1,i}$	L $\bar{d}_{1,i}$	M $\bar{c}_{1,i}$	N $\bar{e}_{2,k}$	O $\bar{e}_{2,k}-\bar{e}_{2}$	P v_k	Q ln(HR)$_k$	R $1/v_k$
	0 to 3	0	100	55	53.5	15.0	3.0	100	98	95.0	39.0	6.0	19.4	-4.5	12.44	-0.36	0.08
	3 to 6	3	72	37	36.5	11.0	1.0	59	53	50.5	12.0	5.0	9.7	1.3	5.61	0.24	0.18
	6 to 9	6	50	25	24.5	4.0	1.0	45	36	31.5	2.0	9.0	2.7	1.4	1.50	0.93	0.67
	9 to 12	9	42	20	17.5	3.0	5.0	42	25	20.0	4.0	10.0	3.3	-0.3	1.74	-0.15	0.57
	12 to 15	12	35	12	7.5	2.0	9.0	34	11	11.0	1.0	0.0	1.2	0.8	0.71	1.09	1.40
	15 to 18	15	26	1	0.5	0.0	1.0	31	10	10.0	0.0	0.0	0.0	0.0	0.00	20.14	472.02
	18 to 21	18	23	0	na	na	na	31	10	8.0	2.0	4.0	na	na	na	na	na
Problem--->	21 to 24	21			na	na	na	23	4	3.5	0.0	1.0	na	na	na	na	na
Problem--->	24 to 27	24			na	na	na	23	3	1.5	0.0	3.0	na	na	na	na	na
Problem--->	27 onwards	27			na	na	na	23	0	na	na	na	na	na	na	na	na
					na	na	na			na	na	na	na	na	na	na	na
					na	na	na			na	na	na	na	na	na	na	na
					na	na	na			na	na	na	na	na	na	na	na
					na	na	na			na	na	na	na	na	na	na	na
					na	na	na			na	na	na	na	na	na	na	na
					na	na	na			na	na	na	na	na	na	na	na
					na	na	na			na	na	na	na	na	na	na	na
					na	na	na			na	na	na	na	na	na	na	na
					na	na	na			na	na	na	na	na	na	na	na
					na	na	na			na	na	na	na	na	na	na	na
					na	na	na			na	na	na	na	na	na	na	na
					na	na	na			na	na	na	na	na	na	na	na
					na	na	na			na	na	na	na	na	na	na	na
					na	na	na			na	na	na	na	na	na	na	na
					na	na	na			na	na	na	na	na	na	na	na

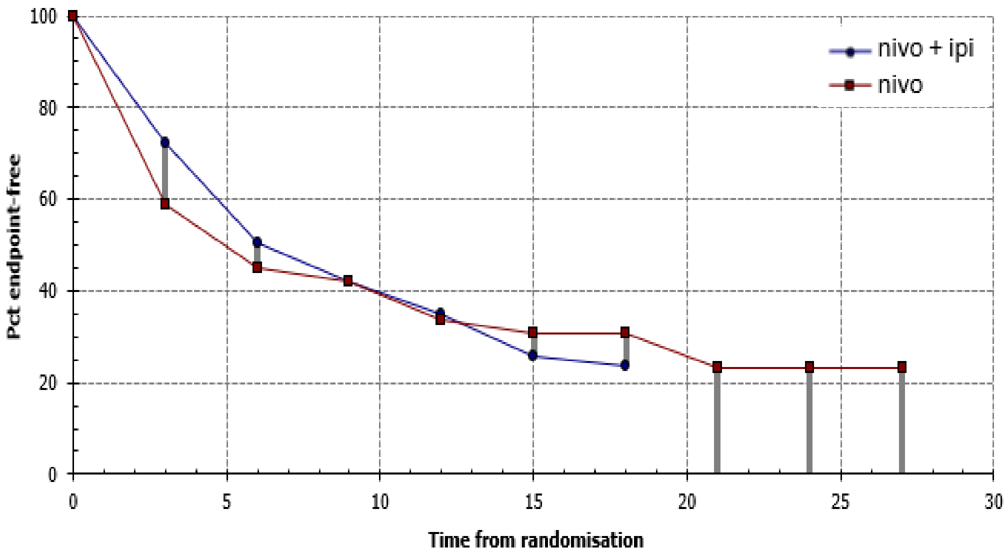

© 2023 Serritella AV et al. *JAMA Oncology*.

D’Angelo et al 2018_ PFS

Key

A: Checks
B: Time interval
C: Start of t

D: Survival prob at t_{i-1} (%)
E: Number alive at t_{i-1}
F: Effective number at risk during t_{i-1},t_i
G: Effective number events during t_{i-1},t_i
H: Effective number censored during t_{i-1},t_i

I: Survival prob at t_{i-1} (%)
J: Number alive at t_{i-1}
K: Effective number at risk during t_{i-1},t_i
L: Effective number events during t_{i-1},t_i
M: Effective number censored during t_{i-1},t_i

N: Proportion of events in research arm
O: Difference in events
P: Variance of the log hazard ratio for t_k
Q: Log hazard ratio for t_k
R: Reciprocal of the variance of the log hazard ratio for t_k

HR: 0.71
ln(HR): -0.35
se(ln(HR)): 0.24

			Research(2): Nivo+Ipi					Control(1): Nivo					Outcomes				
	Total					33.0	8.0				38.0	4.0		-5.8	16.8		
A	B	C	D	E	F	G	H	I	J	K	L	M	N	O	P	Q	R
Checks	$t_{i,i-1}$	t_{i-1}	$s_{2,i}$	$n_{2,i}$	$\bar{n}_{2,i}$	$\bar{d}_{2,i}$	$\bar{c}_{2,i}$	$s_{1,i}$	$n_{1,i}$	$\bar{n}_{1,i}$	$\bar{d}_{1,i}$	$\bar{c}_{1,i}$	$\bar{e}_{2,k}$	$\bar{}_{2,k}-\bar{e}_{2,}$	v_k	$\ln(HR)_k$	$1/v_k$
	0 to 1	0	100	41	41.0	2.0	0.0	100	42	42.0	0.0	0.0	1.0	1.0	0.50	2.02	2.00
	1 to 3	1	95	39	37.5	14.0	3.0	100	42	42.0	26.0	0.0	18.9	-4.9	9.98	-0.49	0.10
	3 to 6	3	60	22	21.5	11.0	1.0	38	16	14.0	8.0	4.0	11.5	-0.5	4.55	-0.11	0.22
	6 to 9	6	29	10	9.5	3.0	1.0	16	4	4.0	2.0	0.0	3.5	-0.5	1.04	-0.49	0.96
	9 to 12	9	20	6	6.0	1.0	0.0	8	2	2.0	1.0	0.0	1.5	-0.5	0.37	-1.29	2.72
	12 to 15	12	16	5	5.0	2.0	0.0	4	1	1.0	1.0	0.0	2.5	-0.5	0.41	-1.23	2.43
	15 to 18	15	10	3	1.5	0.0	3.0	0	0	na	na	na	na	na	na	na	na
Problem--->	18 onwards	18	10	0	na	na	na			na	na	na	na	na	na	na	na
					na	na	na			na	na	na	na	na	na	na	na
					na	na	na			na	na	na	na	na	na	na	na
					na	na	na			na	na	na	na	na	na	na	na
					na	na	na			na	na	na	na	na	na	na	na
					na	na	na			na	na	na	na	na	na	na	na
					na	na	na			na	na	na	na	na	na	na	na
					na	na	na			na	na	na	na	na	na	na	na
					na	na	na			na	na	na	na	na	na	na	na
					na	na	na			na	na	na	na	na	na	na	na
					na	na	na			na	na	na	na	na	na	na	na
					na	na	na			na	na	na	na	na	na	na	na
					na	na	na			na	na	na	na	na	na	na	na
					na	na	na			na	na	na	na	na	na	na	na
					na	na	na			na	na	na	na	na	na	na	na
					na	na	na			na	na	na	na	na	na	na	na
					na	na	na			na	na	na	na	na	na	na	na
					na	na	na			na	na	na	na	na	na	na	na

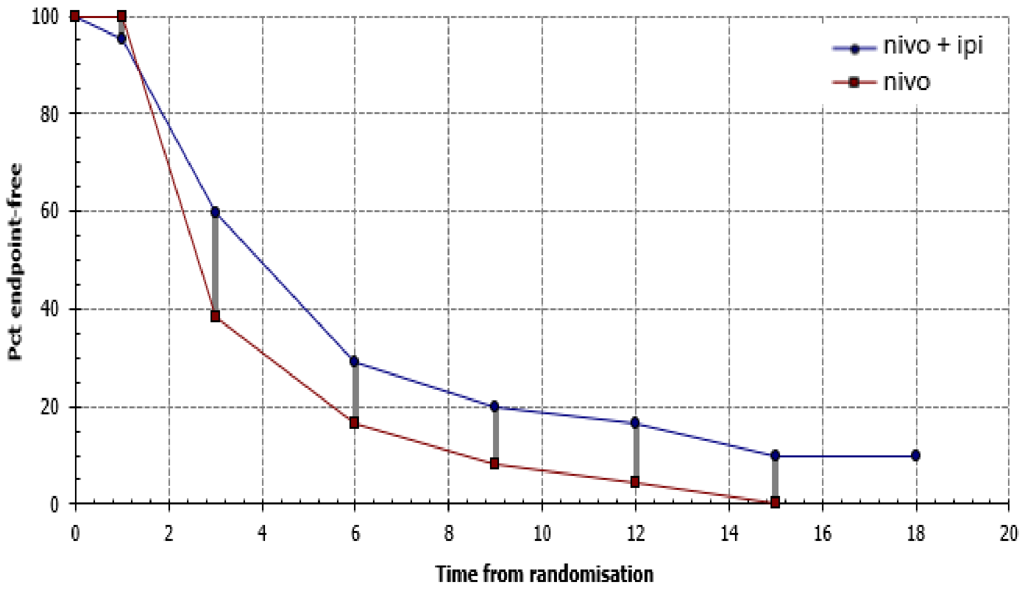

© 2023 Serritella AV et al. *JAMA Oncology*.

D'Angelo et al 2018_ OS

Key

A: Checks
B: Time interval
C: Start of t
D: Survival prob at t_{i-1} (%)
E: Number alive at t_{i-1}
F: Effective number at risk during t_{i-1}, t_i
G: Effective number events during t_{i-1}, t_i
H: Effective number censored during t_{i-1}, t_i
I: Survival prob at t_{i-1} (%)
J: Number alive at t_{i-1}
K: Effective number at risk during t_{i-1}, t_i
L: Effective number events during t_{i-1}, t_i
M: Effective number censored during t_{i-1}, t_i
N: Proportion of events in research arm
O: Difference in events
P: Variance of the log hazard ratio for t_k
Q: Log hazard ratio for t_k
R: Reciprocal of the variance of the log hazard ratio for t_k

HR	0.72
ln(HR)	-0.33
se(ln(HR))	0.29

			Research(2): Nivo+Ipi					Control(1): Nivo					Outcomes				
	Total					21.0	20.0				27.0	15.0		-3.9	11.8		
A	B	C	D	E	F	G	H	I	J	K	L	M	N	O	P	Q	R
Checks	$t_{i,i-1}$	t_{i-1}	$s_{2,i}$	$n_{2,i}$	$n'_{2,i}$	$d'_{2,i}$	$c'_{2,i}$	$s_{1,i}$	$n_{1,i}$	$n'_{1,i}$	$d'_{1,i}$	$c'_{1,i}$	$e'_{2,k}$	$'_{2,k}-e'_{2}$	v_k	$\ln(HR)_k$	$1/v_k$
	0 to 3	0	100	41	41.0	5.0	0.0	100	42	41.5	7.0	1.0	5.9	-0.9	2.99	-0.32	0.33
	3 to 6	3	88	36	35.5	4.0	1.0	83	34	33.5	8.0	1.0	6.1	-2.2	2.98	-0.73	0.34
	6 to 9	6	78	31	30.5	5.0	1.0	63	25	24.0	4.0	2.0	5.0	0.0	2.22	-0.02	0.45
	9 to 12	9	65	25	24.0	3.0	2.0	53	19	17.5	4.0	3.0	4.1	-1.1	1.72	-0.62	0.58
	12 to 15	12	57	20	15.0	4.0	10.0	41	12	10.5	0.0	3.0	2.4	1.6	0.97	1.70	1.03
	15 to 18	15	42	6	3.0	0.0	6.0	41	9	6.5	4.0	5.0	1.3	-1.3	0.87	-1.46	1.15
	18 onwards	18	42	0	na	na	na	16	0	na	na	na	na	na	na	na	na
					na	na	na			na	na	na	na	na	na	na	na
					na	na	na			na	na	na	na	na	na	na	na
					na	na	na			na	na	na	na	na	na	na	na
					na	na	na			na	na	na	na	na	na	na	na
					na	na	na			na	na	na	na	na	na	na	na
					na	na	na			na	na	na	na	na	na	na	na
					na	na	na			na	na	na	na	na	na	na	na
					na	na	na			na	na	na	na	na	na	na	na
					na	na	na			na	na	na	na	na	na	na	na
					na	na	na			na	na	na	na	na	na	na	na
					na	na	na			na	na	na	na	na	na	na	na
					na	na	na			na	na	na	na	na	na	na	na
					na	na	na			na	na	na	na	na	na	na	na
					na	na	na			na	na	na	na	na	na	na	na
					na	na	na			na	na	na	na	na	na	na	na
					na	na	na			na	na	na	na	na	na	na	na
					na	na	na			na	na	na	na	na	na	na	na
					na	na	na			na	na	na	na	na	na	na	na

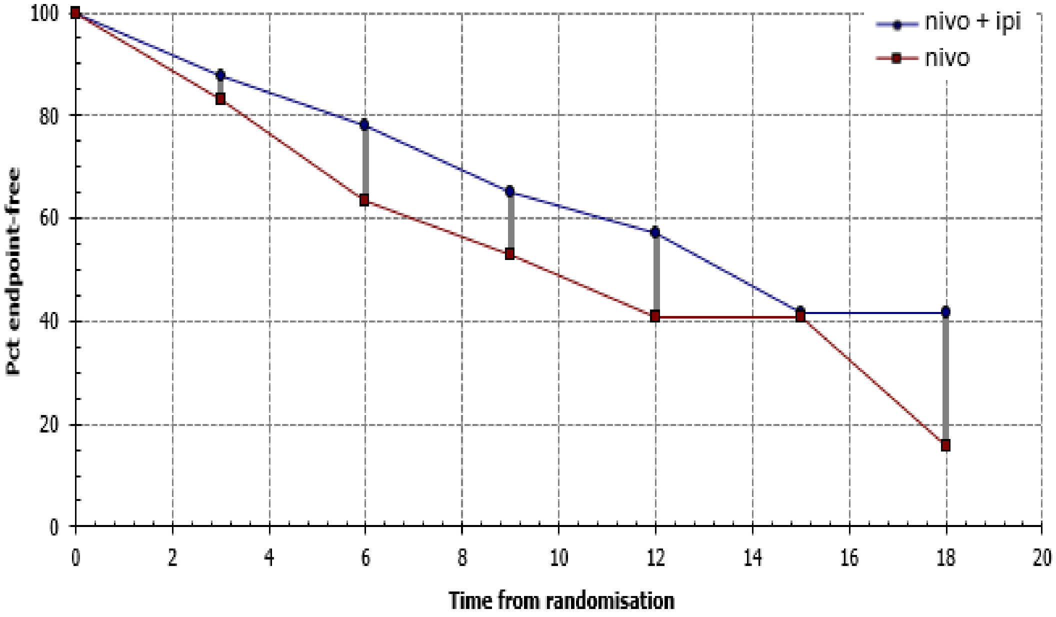

© 2023 Serritella AV et al. *JAMA Oncology*.

Omuro et al 2017_ PFS

Key

A: Checks
B: Time interval
C: Start of t

D: Survival prob at t_{i-1} (%)
E: Number alive at t_{i-1}
F: Effective number at risk during t_{i-1},t_i
G: Effective number events during t_{i-1},t_i
H: Effective number censored during t_{i-1},t_i

I: Survival prob at t_{i-1} (%)
J: Number alive at t_{i-1}
K: Effective number at risk during t_{i-1},t_i
L: Effective number events during t_{i-1},t_i
M: Effective number censored during t_{i-1},t_i

N: Proportion of events in research arm
O: Difference in events
P: Variance of the log hazard ratio for t_k
Q: Log hazard ratio for t_k
R: Reciprocal of the variance of the log hazard ratio for t_k

HR	1.15
ln(HR)	0.14
se(ln(HR))	0.41

			Research(2): Nivo+Ipi					Control(1): Nivo					Outcomes				
	Total					19.0	1.0				8.0	2.0		0.8	5.9		
A Checks	B $t_{i,i-1}$	C t_{i-1}	D $s_{2,i}$	E $n_{2,i}$	F $\bar{n}_{2,i}$	G $\bar{d}_{2,i}$	H $\bar{c}_{2,i}$	I $s_{1,i}$	J $n_{1,i}$	K $\bar{n}_{1,i}$	L $\bar{d}_{1,i}$	M $\bar{c}_{1,i}$	N $\bar{e}_{2,k}$	O $\bar{e}_{2,k}-e_{2}$	P v_k	Q ln(HR)$_k$	R 1/v_k
	0 to 3	0	100	20	20.0	15.0	0.0	100	10	9.5	7.0	1.0	14.9	0.1	4.82	0.01	0.21
	3 to 6	3	25	5	4.5	2.0	1.0	26	2	2.0	1.0	0.0	2.1	-0.1	0.65	-0.10	1.55
	6 to 9	6	14	2	2.0	0.0	0.0	13	1	1.0	0.0	0.0	0.0	0.0	0.00	na	na
	9 to 12	9	14	2	2.0	1.0	0.0	13	1	1.0	0.0	0.0	0.6	0.3	0.22	1.50	4.62
	12 to 15	12	7	1	1.0	0.0	0.0	13	1	1.0	0.0	0.0	0.0	0.0	0.00	na	na
	15 to 18	15	7	1	1.0	0.0	0.0	13	1	1.0	0.0	0.0	0.0	0.0	0.00	na	na
	18 to 21	18	7	1	1.0	0.0	0.0	13	1	1.0	0.0	0.0	0.0	0.0	0.00	na	na
	21 to 24	21	7	1	1.0	0.0	0.0	13	1	1.0	0.0	0.0	0.0	0.0	0.00	na	na
	24 to 27	24	7	1	1.0	1.0	0.0	13	1	1.0	0.0	0.0	0.5	0.5	0.25	2.00	4.00
	27 to 30	27	0	0	0.0	0.0	0.0	13	1	0.5	0.0	1.0	0.0	0.0	0.00	na	na
	30 onwards	30	0	0	na	na	na	13	0	na	na	na	na	na	na	na	na
					na	na	na			na	na	na	na	na	na	na	na
					na	na	na			na	na	na	na	na	na	na	na
					na	na	na			na	na	na	na	na	na	na	na
					na	na	na			na	na	na	na	na	na	na	na
					na	na	na			na	na	na	na	na	na	na	na
					na	na	na			na	na	na	na	na	na	na	na
					na	na	na			na	na	na	na	na	na	na	na
					na	na	na			na	na	na	na	na	na	na	na
					na	na	na			na	na	na	na	na	na	na	na
					na	na	na			na	na	na	na	na	na	na	na
					na	na	na			na	na	na	na	na	na	na	na
					na	na	na			na	na	na	na	na	na	na	na
					na	na	na			na	na	na	na	na	na	na	na
					na	na	na			na	na	na	na	na	na	na	na

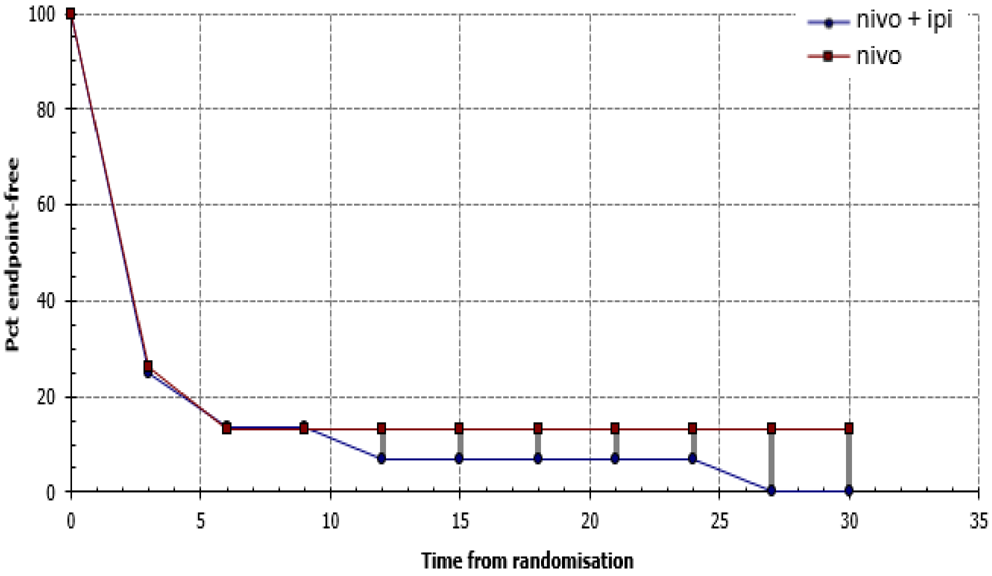

© 2023 Serritella AV et al. *JAMA Oncology*.

Omuro et al 2017_OS

Key

A: Checks
B: Time interval
C: Start of t
D: Survival prob at t_{i-1} (%)
E: Number alive at t_{i-1}
F: Effective number at risk during t_{i-1},t_i
G: Effective number events during t_{i-1},t_i
H: Effective number censored during t_{i-1},t_i
I: Survival prob at t_{i-1} (%)
J: Number alive at t_{i-1}
K: Effective number at risk during t_{i-1},t_i
L: Effective number events during t_{i-1},t_i
M: Effective number censored during t_{i-1},t_i
N: Proportion of events in research arm
O: Difference in events
P: Variance of the log hazard ratio for t_k
Q: Log hazard ratio for t_k
R: Reciprocal of the variance of the log hazard ratio for t_k

**HR**	**1.15**
**ln(HR)**	0.14
**se(ln(HR))**	0.41

			Research(2): Nivo+Ipi					Control(1): Nivo					Outcomes				
	Total					17.0	3.0				9.0	1.0		0.9	6.1		
A Checks	B $t_{i,i-1}$	C t_{i-1}	D $s_{2,i}$	E $n_{2,i}$	F $\bar{n}_{2,i}$	G $\bar{d}_{2,i}$	H $\bar{c}_{2,i}$	I $s_{1,i}$	J $n_{1,i}$	K $\bar{n}_{1,i}$	L $\bar{d}_{1,i}$	M $\bar{c}_{1,i}$	N $\bar{e}_{2,k}$	O $\bar{e}_{2,k}-\bar{e}_{2}$	P v_k	Q $\ln(HR)_k$	R $1/v_k$
	0 to 3	0	100	20	20.0	1.0	0.0	100	10	10.0	0.0	0.0	0.7	0.3	0.22	1.50	4.50
	3 to 6	3	95	19	19.0	8.0	0.0	100	10	10.0	3.0	0.0	7.2	0.8	2.49	0.32	0.40
	6 to 9	6	55	11	11.0	2.0	0.0	70	7	7.0	1.0	0.0	1.8	0.2	0.71	0.23	1.40
	9 to 12	9	45	9	8.5	2.0	1.0	60	6	6.0	2.0	0.0	2.3	-0.3	0.97	-0.35	1.03
	12 to 15	12	34	6	6.0	2.0	0.0	40	4	4.0	1.0	0.0	1.8	0.2	0.72	0.27	1.40
	15 to 18	15	23	4	4.0	1.0	0.0	30	3	3.0	0.0	0.0	0.5	0.4	0.23	1.75	4.29
	18 to 21	18	18	3	3.0	0.0	0.0	30	3	3.0	0.0	0.0	0.0	0.0	0.00	na	na
	21 to 24	21	18	3	3.0	0.0	0.0	30	3	3.0	1.0	0.0	0.5	-0.5	0.25	-2.00	4.00
	24 to 27	24	18	3	3.0	1.0	0.0	20	2	2.0	1.0	0.0	1.2	-0.2	0.49	-0.39	2.05
	27 to 30	27	12	2	1.0	0.0	2.0	10	1	1.0	0.0	0.0	0.0	0.0	0.00	na	na
	30 to 33	30	12	0	0.0	0.0	0.0	10	1	1.0	0.0	0.0	0.0	0.0	0.00	na	na
	33 to 36	33	12	0	0.0	0.0	0.0	10	1	0.5	0.0	1.0	0.0	0.0	0.00	na	na
Problem--->	36 onwards	36		0	na	na	na	10	0	na	na	na	na	na	na	na	na
					na	na	na			na	na	na	na	na	na	na	na
					na	na	na			na	na	na	na	na	na	na	na
					na	na	na			na	na	na	na	na	na	na	na
					na	na	na			na	na	na	na	na	na	na	na
					na	na	na			na	na	na	na	na	na	na	na
					na	na	na			na	na	na	na	na	na	na	na
					na	na	na			na	na	na	na	na	na	na	na
					na	na	na			na	na	na	na	na	na	na	na
					na	na	na			na	na	na	na	na	na	na	na
					na	na	na			na	na	na	na	na	na	na	na
					na	na	na			na	na	na	na	na	na	na	na
					na	na	na			na	na	na	na	na	na	na	na

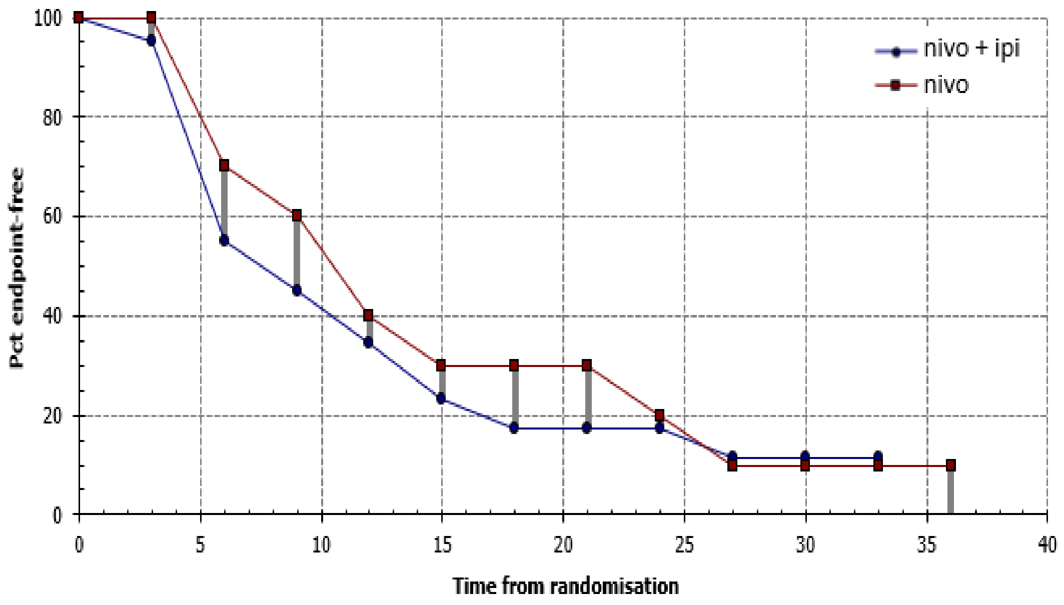

© 2023 Serritella AV et al. *JAMA Oncology*.

eFigure 1. PRISMA Schema

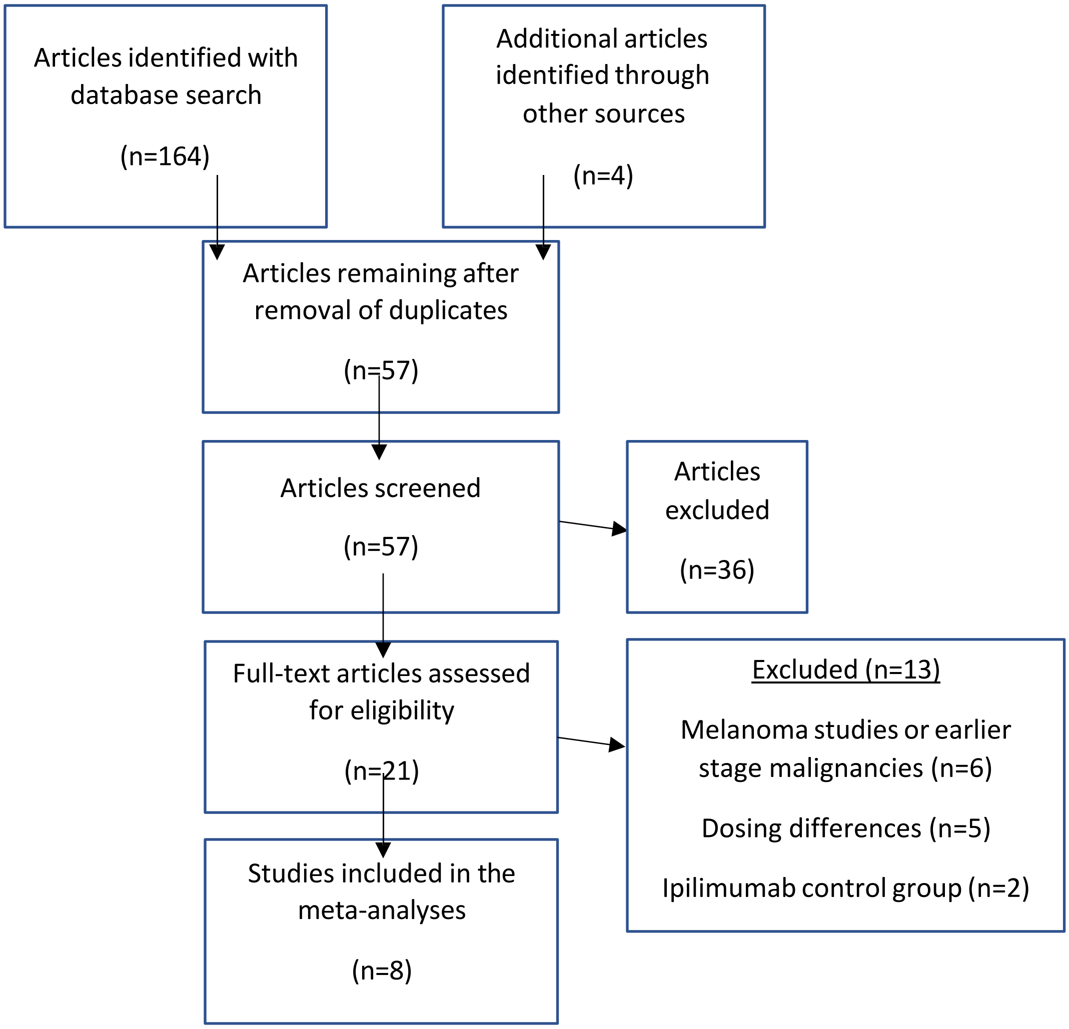

© 2023 Serritella AV et al. *JAMA Oncology*.

eFigure 2. OS and PFS Meta-analysis Without Hellman et al 2019 (Sensitivity Analysis)

Study or Subgroup	log[Hazard Ratio]	SE	Nivo+Ipi Total	Nivo Total	Weight	Hazard Ratio IV, Fixed, 95% CI	Year
Antonia et al 2016	-0.05	0.21	54	98	13.5%	0.95 [0.63, 1.44]	2016
Omuro et al 2017	0.14	0.41	20	10	3.5%	1.15 [0.52, 2.57]	2017
D'Angelo et al 2018	-0.33	0.29	41	42	7.1%	0.72 [0.41, 1.27]	2018
Janjigian et al 2018	0.22	0.21	52	59	13.5%	1.25 [0.83, 1.88]	2018
Hellmann et al 2019	-0.09	0.09	396	396	0.0%	0.91 [0.77, 1.09]	2019
Scherpereel et al 2019	-0.28	0.23	62	63	11.3%	0.76 [0.48, 1.19]	2019
Sharma et al 2019	0.18	0.17	104	78	20.6%	1.20 [0.86, 1.67]	2019
Gettinger et al 2021	-0.12	0.14	125	127	30.4%	0.89 [0.67, 1.17]	2021
**Total (95% CI)**			**458**	**477**	**100.0%**	**0.97 [0.84, 1.13]**	

Heterogeneity: Chi² = 5.79, df = 6 (P = 0.45); I² = 0%
Test for overall effect: Z = 0.34 (P = 0.73)

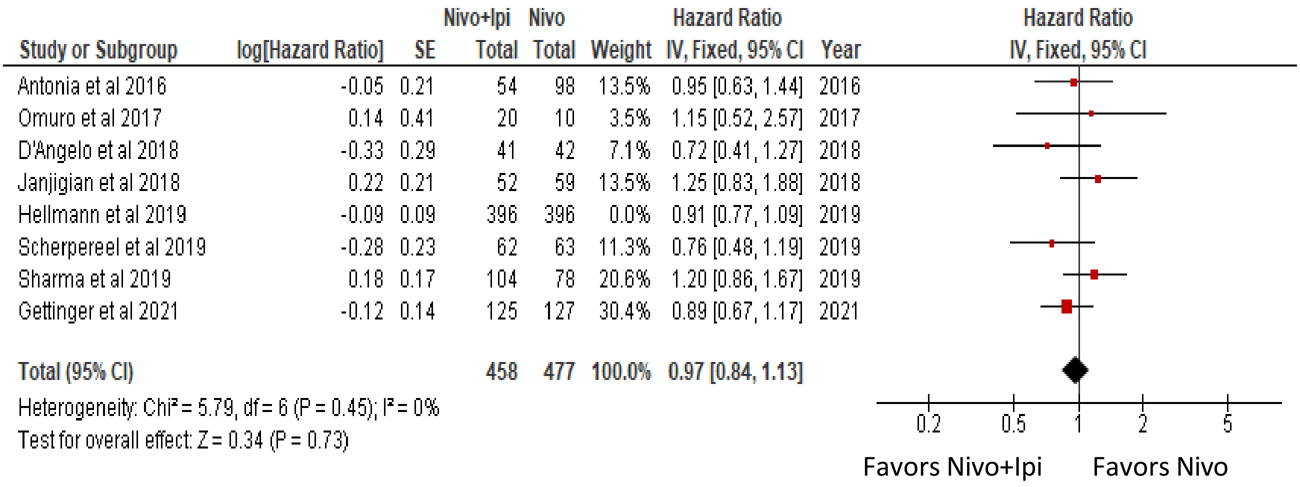

Study or Subgroup	log[Hazard Ratio]	SE	Nivo+Ipi Total	Nivo Total	Weight	Hazard Ratio IV, Fixed, 95% CI	Year
Antonia et al 2016	-0.01	0.19	54	98	13.5%	0.99 [0.68, 1.44]	2016
Omuro et al 2017	0.14	0.41	20	10	2.9%	1.15 [0.52, 2.57]	2017
D'Angelo et al 2018	-0.34	0.24	41	42	8.5%	0.71 [0.44, 1.14]	2018
Janjigian et al 2018	0.01	0.2	52	59	12.2%	1.01 [0.68, 1.49]	2018
Hellmann et al 2019	-0.17	0.08	396	396	0.0%	0.84 [0.72, 0.99]	2019
Scherpereel et al 2019	-0.16	0.18	62	63	15.0%	0.85 [0.60, 1.21]	2019
Sharma et al 2019	0.03	0.16	104	78	19.0%	1.03 [0.75, 1.41]	2019
Gettinger et al 2021	-0.18	0.13	125	127	28.8%	0.84 [0.65, 1.08]	2021
**Total (95% CI)**			**458**	**477**	**100.0%**	**0.91 [0.79, 1.04]**	

Heterogeneity: Chi² = 3.01, df = 6 (P = 0.81); I² = 0%
Test for overall effect: Z = 1.36 (P = 0.17)

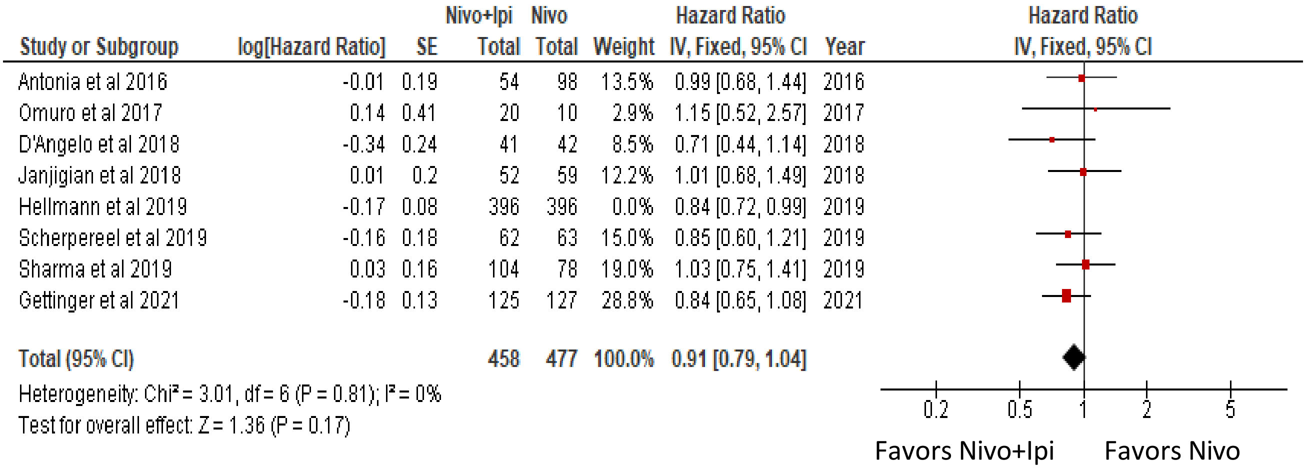

© 2023 Serritella AV et al. *JAMA Oncology*.

eFigure 3. Grade 3 to 4 Hepatotoxicity

Study or Subgroup	Nivo+Ipi Events	Nivo+Ipi Total	Nivo Events	Nivo Total	Weight	Odds Ratio M-H, Random, 95% CI	Year
Antonia et al 2016	0	54	0	98		Not estimable	2016
Omuro et al 2017	2	20	0	10	3.1%	2.84 [0.12, 64.87]	2017
D'Angelo et al 2018	5	41	0	42	3.6%	12.81 [0.68, 239.54]	2018
Janjigian et al 2018	3	52	5	59	12.6%	0.66 [0.15, 2.91]	2018
Scherpereel et al 2019	8	62	0	63	3.7%	19.81 [1.12, 351.11]	2019
Sharma et al 2019	7	104	2	78	11.0%	2.74 [0.55, 13.58]	2019
Hellmann et al 2019	47	396	15	396	47.3%	3.42 [1.88, 6.23]	2019
Gettinger et al 2021	11	125	4	127	18.8%	2.97 [0.92, 9.58]	2021
**Total (95% CI)**		**854**		**873**	**100.0%**	**2.94 [1.67, 5.15]**	
Total events	83		26				

Heterogeneity: $Tau^2 = 0.08$; $Chi^2 = 6.88$, df = 6 (P = 0.33); $I^2 = 13\%$

Test for overall effect: Z = 3.76 (P = 0.0002)

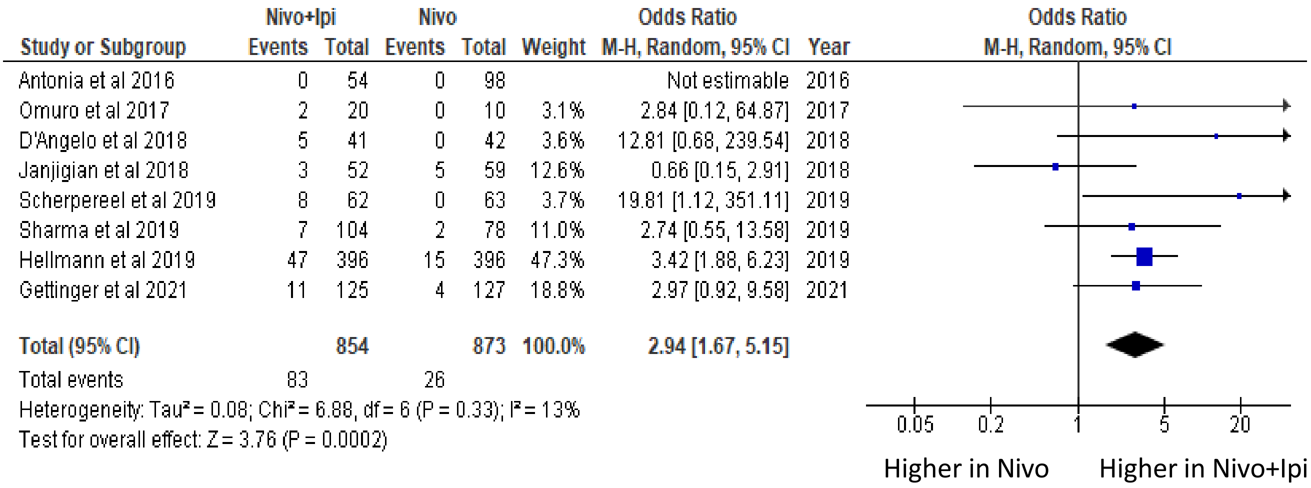

© 2023 Serritella AV et al. *JAMA Oncology*.

eFigure 4. Grade 3 to 4 GI Toxicity

Study or Subgroup	Nivo-Ipi Events	Nivo-Ipi Total	Nivo Events	Nivo Total	Weight	Odds Ratio M-H, Fixed, 95% CI	Year
Antonia et al 2016	3	54	0	98	3.2%	13.39 [0.68, 264.19]	2016
Omuro et al 2017	2	20	0	10	5.5%	2.84 [0.12, 64.87]	2017
Janjigian et al 2018	1	52	1	59	8.7%	1.14 [0.07, 18.65]	2018
D'Angelo et al 2018	0	41	1	42	13.8%	0.33 [0.01, 8.42]	2018
Sharma et al 2019	8	104	1	78	9.9%	6.42 [0.79, 52.42]	2019
Hellmann et al 2019	14	396	4	396	36.4%	3.59 [1.17, 11.01]	2019
Scherpereel et al 2019	2	62	0	63	4.5%	5.25 [0.25, 111.56]	2019
Gettinger et al 2021	4	125	2	127	18.1%	2.07 [0.37, 11.49]	2021
**Total (95% CI)**		**854**		**873**	**100.0%**	**3.28 [1.65, 6.49]**	
Total events	34		9				

Heterogeneity: Chi² = 4.13, df = 7 (P = 0.77); I² = 0%

Test for overall effect: Z = 3.41 (P = 0.0007)

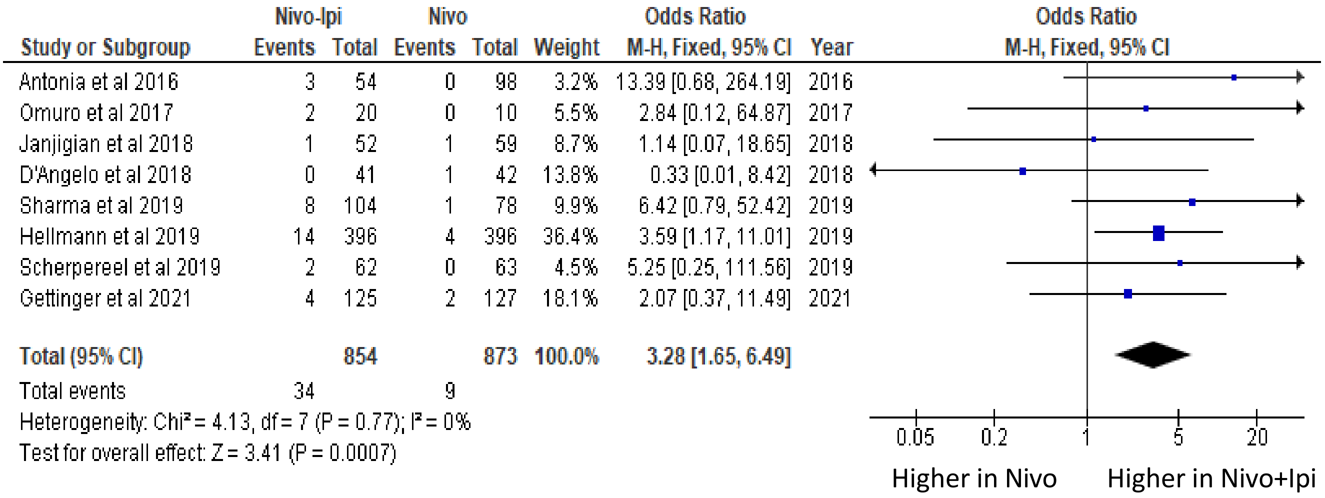

© 2023 Serritella AV et al. *JAMA Oncology*.

eFigure 5. Grade 3 to 4 Pneumonitis

	Nivo-Ipi		Nivo			Odds Ratio		Odds Ratio
Study or Subgroup	Events	Total	Events	Total	Weight	M-H, Random, 95% CI	Year	M-H, Random, 95% CI
Antonia et al 2016	1	54	1	98	5.4%	1.83 [0.11, 29.86]	2016	
Omuro et al 2017	0	20	0	10		Not estimable	2017	
Janjigian et al 2018	0	52	0	59		Not estimable	2018	
D'Angelo et al 2018	0	41	0	42		Not estimable	2018	
Hellmann et al 2019	19	396	6	396	49.0%	3.28 [1.29, 8.29]	2019	
Scherpereel et al 2019	1	62	0	63	4.1%	3.10 [0.12, 77.51]	2019	
Sharma et al 2019	1	104	0	78	4.1%	2.28 [0.09, 56.61]	2019	
Gettinger et al 2021	9	125	6	127	37.4%	1.56 [0.54, 4.53]	2021	
**Total (95% CI)**		**854**		**873**	**100.0%**	**2.37 [1.24, 4.54]**		
Total events	31		13					

Heterogeneity: Tau² = 0.00; Chi² = 1.12, df = 4 (P = 0.89); I² = 0%

Test for overall effect: Z = 2.60 (P = 0.009)

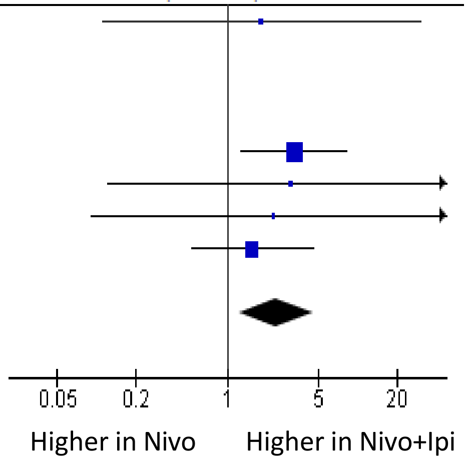

© 2023 Serritella AV et al. *JAMA Oncology*.

eFigure 6. Grade 3 to 4 Endocrine Dysfunction

Study or Subgroup	Nivo-Ipi Events	Nivo-Ipi Total	Nivo Events	Nivo Total	Weight	Odds Ratio M-H, Random, 95% CI	Year
Antonia et al 2016	0	54	0	98		Not estimable	2016
Omuro et al 2017	0	20	0	10		Not estimable	2017
D'Angelo et al 2018	1	41	0	42	12.3%	3.15 [0.12, 79.54]	2018
Janjigian et al 2018	0	52	0	59		Not estimable	2018
Sharma et al 2019	3	104	0	78	14.4%	5.41 [0.28, 106.35]	2019
Hellmann et al 2019	24	396	2	396	60.9%	12.71 [2.98, 54.15]	2019
Scherpereel et al 2019	1	62	0	63	12.3%	3.10 [0.12, 77.51]	2019
Gettinger et al 2021	0	125	0	127		Not estimable	2021
**Total (95% CI)**		**854**		**873**	**100.0%**	**7.95 [2.57, 24.65]**	
Total events	29		2				

Heterogeneity: Tau² = 0.00; Chi² = 1.14, df = 3 (P = 0.77); I² = 0%

Test for overall effect: Z = 3.59 (P = 0.0003)

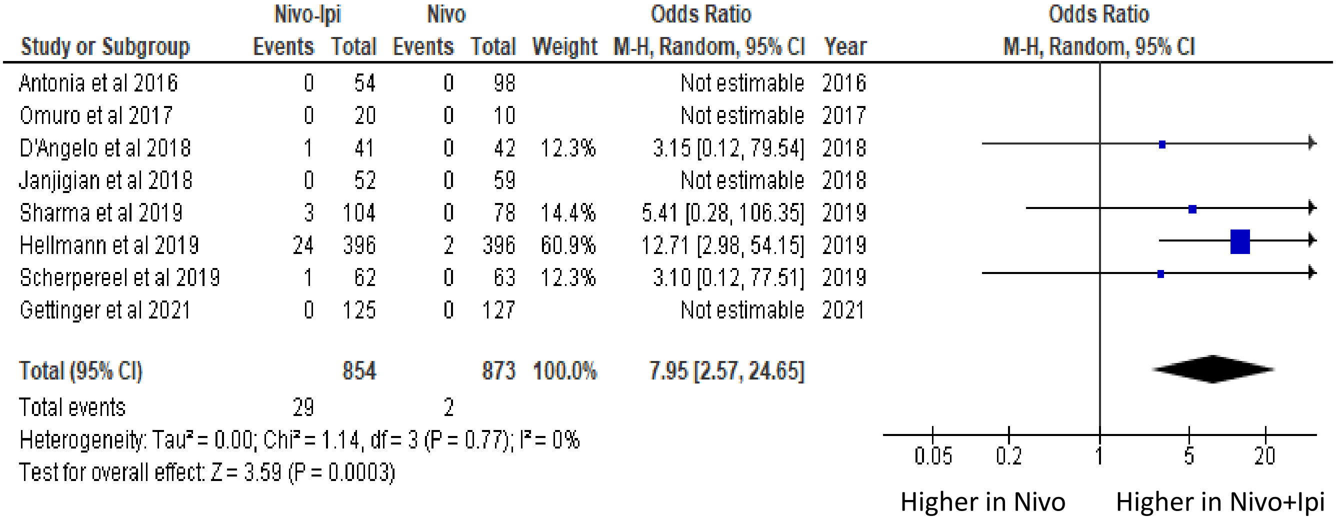

© 2023 Serritella AV et al. *JAMA Oncology*.

eFigure 7. Grade 3 to 4 Fatigue

Study or Subgroup	Nivo+Ipi Events	Nivo+Ipi Total	Nivo Events	Nivo Total	Weight	Odds Ratio M-H, Fixed, 95% CI	Year
Antonia et al 2016	0	54	1	98	6.9%	0.60 [0.02, 14.89]	2016
Omuro et al 2017	3	20	0	10	3.6%	4.20 [0.20, 89.61]	2017
D'Angelo et al 2018	2	41	1	42	6.1%	2.10 [0.18, 24.13]	2018
Janjigian et al 2018	0	52	1	59	9.1%	0.37 [0.01, 9.32]	2018
Hellmann et al 2019	8	396	2	396	12.7%	4.06 [0.86, 19.25]	2019
Scherpereel et al 2019	3	62	1	63	6.1%	3.15 [0.32, 31.16]	2019
Sharma et al 2019	3	104	2	78	14.4%	1.13 [0.18, 6.92]	2019
Gettinger et al 2021	11	125	7	127	41.1%	1.65 [0.62, 4.41]	2021
**Total (95% CI)**		**854**		**873**	**100.0%**	**1.91 [1.03, 3.52]**	
Total events	30		15				

Heterogeneity: Chi² = 3.25, df = 7 (P = 0.86); I² = 0%

Test for overall effect: Z = 2.05 (P = 0.04)

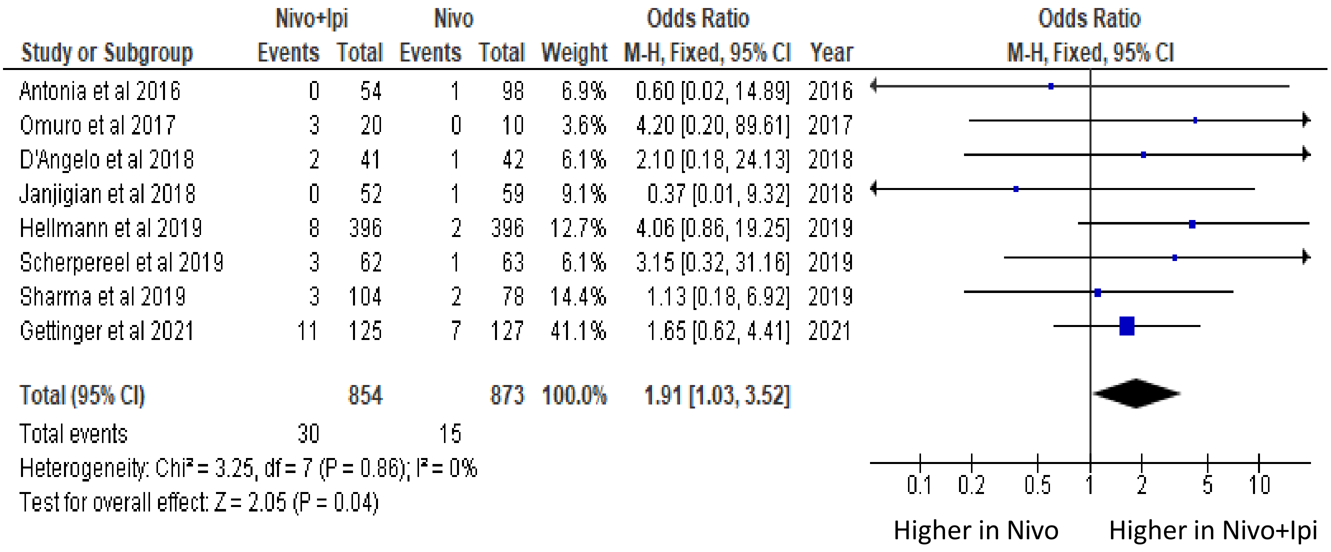

© 2023 Serritella AV et al. *JAMA Oncology*.

eFigure 8. Grade 3 to 4 Dermatitis

	Nivo-Ipi		Nivo			Odds Ratio	
**Study or Subgroup**	**Events**	**Total**	**Events**	**Total**	**Weight**	**M-H, Random, 95% CI**	**Year**
Antonia et al 2016	0	54	0	98		Not estimable	2016
Omuro et al 2017	0	20	0	10		Not estimable	2017
Janjigian et al 2018	0	52	0	59		Not estimable	2018
D'Angelo et al 2018	0	41	0	42		Not estimable	2018
Hellmann et al 2019	24	396	4	396	40.0%	6.32 [2.17, 18.40]	2019
Scherpereel et al 2019	1	62	0	63	12.7%	3.10 [0.12, 77.51]	2019
Sharma et al 2019	2	104	3	78	26.7%	0.49 [0.08, 3.01]	2019
Gettinger et al 2021	3	125	1	127	20.6%	3.10 [0.32, 30.20]	2021
**Total (95% CI)**		**854**		**873**	**100.0%**	**2.52 [0.68, 9.36]**	
Total events	30		8				

Heterogeneity: Tau² = 0.83; Chi² = 5.70, df = 3 (P = 0.13); I² = 47%
Test for overall effect: Z = 1.38 (P = 0.17)

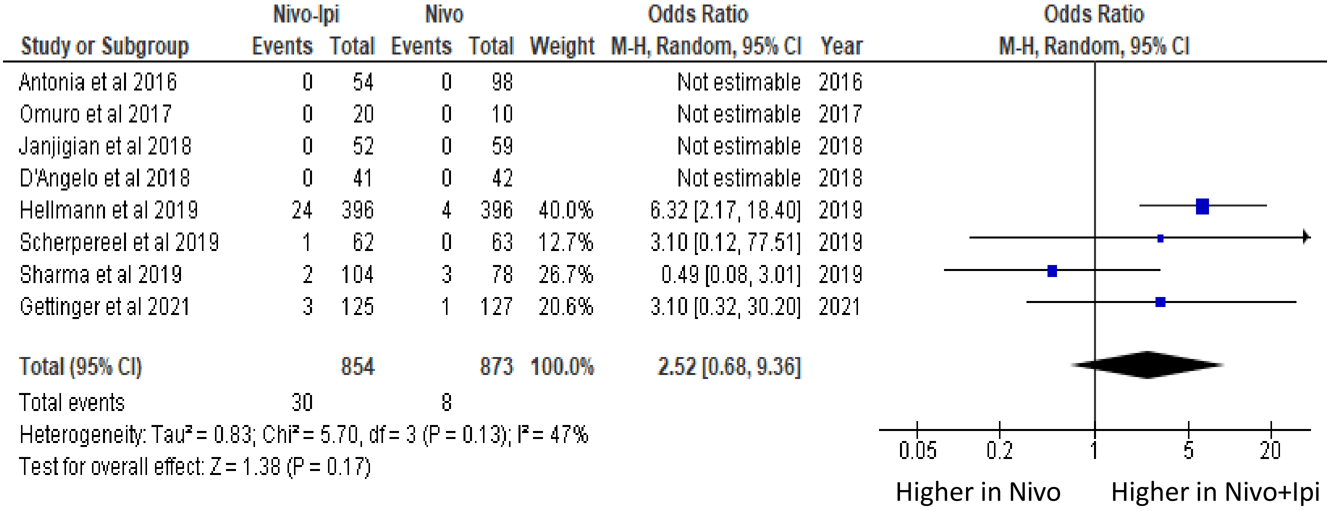

© 2023 Serritella AV et al. *JAMA Oncology*.
