# Supplementary material for: Nivolumab Plus Ipilimumab vs Nivolumab Alone in Advanced Cancers Other Than Melanoma: A Meta-Analysis
Source: JAMA Oncol. 2023 Aug 31;9(10):1441–6. doi: 10.1001/jamaoncol.2023.3295 (PMC10472261; doi:10.1001/jamaoncol.2023.3295)
Supplement: Supplement 2. — Data Sharing Statement [file jamaoncol-e233295-s002.pdf]

Data Sharing Statement

Serritella. Nivolumab Plus Ipilimumab vs Nivolumab Alone in Advanced Cancers Other Than Melanoma. *JAMA Oncol*. Published August 31, 2023. doi:10.1001/jamaoncol.2023.3295

Data
Data available: No

Additional Information
Explanation for why data not available: This is a meta-analysis that utilized the published data of included studies. The detailed calculations of OS and PFS HR estimation for individual studies based on summary statistics of K-M curves have been provided as a separate supplemental file
